# Acute Exercise Leads to Regulation of Telomere-Associated Genes and MicroRNA Expression in Immune Cells

**DOI:** 10.1371/journal.pone.0092088

**Published:** 2014-04-21

**Authors:** Warrick L. Chilton, Francine Z. Marques, Jenny West, George Kannourakis, Stuart P. Berzins, Brendan J. O’Brien, Fadi J. Charchar

**Affiliations:** 1 School of Health Sciences, Federation University Australia, Victoria, Australia; 2 Fiona Elsey Cancer Research Institute, Ballarat, Victoria, Australia; University of Newcastle, United Kingdom

## Abstract

Telomeres are specialized nucleoprotein structures that protect chromosomal ends from degradation. These structures progressively shorten during cellular division and can signal replicative senescence below a critical length. Telomere length is predominantly maintained by the enzyme telomerase. Significant decreases in telomere length and telomerase activity are associated with a host of chronic diseases; conversely their maintenance underpins the optimal function of the adaptive immune system. Habitual physical activity is associated with longer leukocyte telomere length; however, the precise mechanisms are unclear. Potential hypotheses include regulation of telomeric gene transcription and/or microRNAs (miRNAs). We investigated the acute exercise-induced response of telomeric genes and miRNAs in twenty-two healthy males (mean age = 24.1±1.55 years). Participants undertook 30 minutes of treadmill running at 80% of peak oxygen uptake. Blood samples were taken before exercise, immediately post-exercise and 60 minutes post-exercise. Total RNA from white blood cells was submitted to miRNA arrays and telomere extension mRNA array. Results were individually validated in white blood cells and sorted T cell lymphocyte subsets using quantitative real-time PCR (qPCR). Telomerase reverse transcriptase (*TERT*) mRNA (*P = *0.001) and sirtuin-6 (*SIRT6*) (*P*<0.05) mRNA expression were upregulated in white blood cells after exercise. Fifty-six miRNAs were also differentially regulated post-exercise (FDR <0.05). *In silico* analysis identified four miRNAs (miR-186, miR-181, miR-15a and miR-96) that potentially targeted telomeric gene mRNA. The four miRNAs exhibited significant upregulation 60 minutes post-exercise (*P*<0.001). Telomeric repeat binding factor 2, interacting protein (*TERF2IP*) was identified as a potential binding target for miR-186 and miR-96 and demonstrated concomitant downregulation (*P*<0.01) at the corresponding time point. Intense cardiorespiratory exercise was sufficient to differentially regulate key telomeric genes and miRNAs in white blood cells. These results may provide a mechanistic insight into telomere homeostasis and improved immune function and physical health.

## Introduction

There is mounting evidence of an association between habitual physical activity and longer leukocyte telomere length (LTL) [1]–[6]. Telomeres are specialized nucleoprotein structures that protect the ends of linear chromosomes and progressively shorten with each round of cellular division. At a critically shortened threshold, genomic instability, replicative senescence and apoptosis ensue. Accelerated telomere shortening is associated with a host of age-related chronic diseases and risk factors [7]–[15]. Adaptive immune cells regulate telomere length via the enzyme telomerase. Telomerase minimally consists of two core components; the catalytic subunit, telomerase reverse transcriptase (TERT) and an antisense RNA template (TERC). The shelterin complex, a dynamic conglomerate of six telomeric accessory proteins also plays a major role in telomere homeostasis [16]. White blood cells (WBCs) were chosen for telomeric gene analysis due to the high correlation between telomere length in these cells and those of other tissue types [17]–[20].

It is unknown whether the association between physical activity and telomere length in WBCs is due to the amelioration of oxidative stress and inflammation, the exercise-induced regulation of telomeric genes or a complex interplay between all three. Another possible mechanism is the differential regulation of microRNAs (miRNAs), which are known to respond acutely to physical exercise [21]–[24]. miRNAs are short, non-coding RNA molecules which post-transcriptionally regulate gene expression by binding to the 3′ or 5′ untranslated regions (UTR) of messenger RNA (mRNA). Despite burgeoning roles for miRNAs as potential mediators of exercise-induced adaptive processes, little is known about miRNA involvement in telomeric homeostasis. Epigenetic modifications play a key role in telomere length homeostasis [25]–[27], hTERT and telomerase regulation [28]–[30]; however, little is known about the role of miRNA-mediated regulation of telomeric genes.

The aim of this study was to investigate potential mechanisms underpinning the positive association between physical activity and WBC telomere length. The specific aims were to investigate the acute effects of 30 minutes of intense cardiorespiratory exercise on the expression of genes involved in telomere regulation in WBCs, and to identify the exercise-induced expression patterns of miRNAs with potential telomeric involvement.

## Methods

### Ethics Statement

All eligible participants read a plain language information statement outlining all aspects of the project in lay terminology. Informed consent documents explaining the purpose, potential risks and benefits of the project were then signed in the presence of a witness. The study, recruitment and consent procedures were approved by the Human Research Ethics Committee from Federation University Australia.

### Participants

Twenty two healthy, non-smoking males (mean age = 24.1±1.5 years) were recruited to participate in this study (Table 1). Specific health and medical history was obtained via a physical activity readiness questionnaire (PAR-Q) and general lifestyle information was obtained via a health and lifestyle survey.

**Table 1:** Physiological characteristics of the 22 male Participants.

Characteristic	Mean	SD
Age (years)	24.0	±7.3
Height (cm)	180.7	±4.3
Body Mass (kg)	78.5	±9.0
BMI (kg/m^2^)	24.0	±2.5
Waist (cm)	81.5	±5.6
Hip (cm)	98.6	±5.1
Waist:hip ratio	0.8	±0.03
Systolic BP (mmHg)	130.8	±11.7
Diastolic BP (mmHg)	72.0	±8.3
Resting HR (b^.^min^−1^)	64.1	±11.6

SD (standard deviation); BMI (body mass index); BP (blood pressure); mmHg (millimetres of mercury).

### Physiological Measurements

Participants were seated comfortably for 10–15 minutes prior to resting blood pressure and heart rate measurements. Standard, calibrated electronic scales were used to determine body mass and a standard free-standing stadiometer was used to determine height. Waist and hip measurements were taken at standardized sites using a 2 m metal anthropometry tape measure.

### Fitness Measurement

Participants undertook a treadmill-based peak oxygen uptake (V·O_2peak_) test using a Metalyser metabolic system (Cortex Biophysic, Leipzig, Germany). The test protocol started with a five minute warm up period at 10 km^.^h^−1^ after which the speed increased by 1 km^.^h^−1^ each minute. The incline remained at a constant 0% throughout the test. Breath by breath gas exchange and heart rate were continually monitored. The test was terminated when the participant indicated volitional failure and/or when oxygen dynamics showed obvious levelling-off despite increases in work rate.

### Exercise Protocol

A minimum of five days and no more than seven days after the fitness test, each participant undertook the exercise intervention. Participants were asked to refrain from vigorous physical activity during the preceding 48 hours. A 10 ml resting blood sample was taken from the median cubital vein before the exercise bout. Participants then rested for 30 minutes before undertaking 30 minute continuous bout of treadmill running at 80% of previously determined O_2peak_. Breath by breath gas analysis was conducted throughout to ensure the appropriate intensity was maintained.

**Figure 1:**
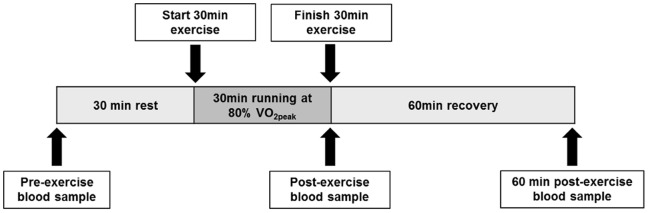
A schematic overview of participant blood sampling and exercise intervention. A baseline blood sample was taken 30 minutes before the onset of exercise. Participants then completed a 30 minute bout of treadmill running at 80% of previously determined V·O_2peak._ Additional blood samples were taken immediately post-exercise and at 60 minutes post-exercise.

Participants undertook a second blood test immediately after the exercise intervention and a third and final blood test 60 minutes after completing the exercise bout (Figure 1). Participants were instructed not to consume caffeine, alcohol or nicotine between the second and final blood tests. Blood was collected using 10 ml K2E EDTA Vacutainer blood collection tubes with BD Vacutainer Eclipse Blood Collection Needles (BD Biosciences). Samples were kept on ice until WBC isolation and storage. All testing was conducted between 7∶30 am to 10∶30 am to limit circadian influence. The time from blood draw to white blood cell isolation never exceeded 90 minutes.

### Preparation of Cells

Whole blood samples were spun at 1000×g (2250 rpm) (4°C) for 15 minutes to separate the plasma and haematocrit sub-fractions. The isolated buffy coats were removed and treated with red blood cell lysis buffer, spun at 300×g for 10 minutes and washed twice in sterile phosphate buffered saline (PBS). This whole blood separation technique isolated WBCs, a heterogeneous mix of neutrophils, basophils, eosinophils, lymphocytes and monocytes. The WBCs were re-suspended in Iscove’s Modified Dulbecco’s Medium (Life Technologies) containing 10% Fetal Bovine Serum (FBS) (Life Technologies) and 200 µl (10% of total end volume) of Dimethyl Sulfoxide (DMSO) (Sigma-Aldrich). Each sample was stored at −80°C for 24 hours before being transferred to liquid nitrogen storage.

Frozen cells were thawed and incubated for 30 minutes at 4°C with the following monoclonal antibodies (mAbs): anti-CD3-FITC, anti-CD4-V450, anti-CD8-APC, anti-CD45RA-PEcy7 and anti-CD45RO-PE (BD Biosciences). The cells were then washed twice with PBS with 1% fetal bovine serum (PBS/FBS) and re-suspended in 200 µl of PBS/FBS and 5 µl of Propidium iodide (PI) prior to analysis. All mAbs were titrated to determine optimal concentration. Pre-acquisition compensation using single stains and fluorescence minus one stains was conducted to remove overlapping fluorescence emission spectra.

### Flow Cytometry

Flow cytometry was performed using a FACSARIA II Flow Cytometer (BD Biosciences), utilizing a red laser emitting at 633-nm, a blue laser emitting at 488-nm and a violet laser emitting at 405-nm. Total lymphocytes were electronically gated based on forward scatter (FSC-A)/side scatter (SSC-A) distribution. Live lymphocytes were gated according to their expression of PI against FSC-A. The CD3+ T cell population was identified by FSC-A against FITC expression and then further separated into CD3+CD4+ and CD3+CD8+ subsets based on positive antigen expression. The CD45RA+ (naïve) and CD45RO+ (memory) subsets were identified based on differential expression of CD45RA and CD45RO. Cells exclusively gated on positive expression of CD45RA will encompass naïve and CD45RA+ effector memory phenotypes in both CD4+ and CD8+ subsets. Similarly, exclusively gating on positive expression of CD45RO will encompass central memory and effector memory phenotypes [31]. Sorted cells were sorted into PBS and stored at −80°C. All data were processed using FlowJo flow cytometry analysis software (Tree Star).

### RNA Extraction

Total RNA was extracted using TRIzol (Life Technologies) according manufacturer’s instructions. All RNA samples were quantified by spectrophotometry using a Nanodrop (Thermo Fisher).

### miRNA Expression Microarrays

Genome-wide miRNA expression arrays (Agilent Human miRNA Microarray, Release 19.0) were performed on pre-exercise and immediately post-exercise samples from a subset of 10 male participants closely matched for age, BMI and V·O_2peak_. The microarrays were performed at the Ramaciotti Centre for Gene Function Analysis (University of New South Wales, Sydney, Australia), as previously described [32]. The data set obtained has been deposited in the NCBI Gene Expression Omnibus database according to the *Minimum Information About a Microarray Experiment (MIAME)* guidelines [33], with series accession number GSE45041.

### Selection and Validation of Candidate miRNAs

Differentially expressed miRNAs were further analysed for potential binding to telomeric gene transcripts using *miRGEN Targets* (http://www.diana.pcbi.upenn.edu/cgi-bin/miRGen/v3/Targets.cgi) [34], which simultaneously collates and analyses intersections and unions of prominent *in silico* analyses such as PicTar, miRagen, microRNA.org, miRanda and TargetScan. Four miRNAs: miR-181b, miR-186, miR-15a and miR-96 were selected for individual validations via qPCR based on predicted interactions between the following miRNAs and telomeric gene transcripts: miR-181b and *TERT*, miR-186 and *TERF2IP, RAD50* and *SIRT6*, miR-96 and *TERF2IP* and miR-15a and TATA box binding protein (*TBP*) (Table S1).

TaqMan assays (Life Technologies) were used to validate the target miRNAs in 18 male participants at the pre- and post-exercise time-points in accordance with the miRNA expression arrays (Table S2). Additionally the target miRNAs were assessed in the same 18 males at the 60 min post-exercise time-point. Briefly, 250 ng of total RNA was reverse transcribed for primers using TaqMan MicroRNA Reverse Transcription kit (Life Technologies) according to manufacturer’s instructions. All reactions were performed in a BioRad thermocycler (BioRad).

The qPCR reactions were performed in duplicate in a Viia7 Real-Time PCR System (Applied Biosystems). All reactions were normalized to the average of RNU44 and RNU48; both of which have been used extensively as endogenous controls in exercise and immunological studies [21], [22], [35]. Validations were also performed in pooled CD4+CD45RA+ T cells, CD4+CD45RO+ T cells, CD8+CD45RA+ T cells and CD8+CD45RO+ T cells using the above method.

### Global Expression of Telomere Extension Genes

To assess the acute effects of exercise on a wide range of telomeric genes, pooled WBC RNA from each time point was analysed using a TaqMan Array Human - Telomere Extension by Telomerase (Life Technologies). Each plate contained 28 assays specific to telomere extension by telomerase associated genes and four assays to candidate endogenous control genes; all reactions were performed in triplicate. Genes were selected for individual sample validation based on fold difference between the three time-points.

### Validation of Candidate Telomeric Genes

Candidate telomeric genes were assessed at three time points via qPCR. Total RNA was reverse transcribed using the Applied Biosystems High Capacity Reverse Transcription Kit (Life Technologies). The qPCR reactions were performed for *TERT*, sirtuin 6 (*SIRT6*), RAD50 homolog (S. cerevisiae) (*RAD50*) and telomeric repeat binding factor 2, interacting protein (*TERF2IP*, also known as *RAP1*) in a Viia7 PCR System (Life Technologies). Details of qPCR primers are listed in Tables S3 and S4. Target genes were normalized to glyceraldehyde-3-phosphate dehydrogenase (*GAPDH*), and analysed using the 2^−ΔΔ^Ct method [36].

### Pooled T Cell Subsets

To identify transcriptionally responsive subsets, CD45RA+ and CD45RO+ subsets in both CD4+ and CD8+ T cells were assessed at each time point. A total of 100 ng of total RNA was pooled from each cell population at each time point from 22 male participants. Sample pooling identifies the transcriptional characteristics of specific cell populations as opposed to individuals and reduces the effects of biological variation. The small RNA yield from the sorted cell subpopulations precluded large numbers of individual samples. Whilst this technique restricts the scope of stringent statistical analysis, it does provide a transcriptional profile that can be compared to unsorted WBCs. Reverse transcription and qPCR reactions were performed according to previously outlined protocols.

### Statistical Analysis

miRNA microarray samples were between-array normalized using the quantile method in Partek Genomics Suite (version 6.6). Differentially expressed miRNAs were identified using a paired t-test false discovery rate (FDR) <0.05. qPCR data were assessed using Friedman’s repeated measures for non-parametric data and repeated measures ANOVA with post-hoc for parametric data. Statistical significance was set at *P*<0.05. All statistical analysis was performed using SPSS Version 17.

## Results

### Exercise Testing and Intervention

The treadmill test duration was 12.5±1.6 min:s. The O_2_ peak achieved was 49.3±4.7 mL⋅kg⋅min^−1^; corresponding to the 75^th^ percentile of maximal aerobic power for males aged 20–29 years. The maximal respiratory exchange ratio (RER) achieved during the treadmill test was 1.2±0.1. The work rate performed during the the 30 minutes of treadmill running corresponded to 80.8±7.1% of O_2_ peak and the average RER was 1.0±0.1. A summary of the treadmill ramp test and 30 minute exercise intervention data appears in Table 2.

**Table 2:** Treadmill ramp test and exercise intervention data.

Treadmill ramp test data	Mean		SD
O_2_ peak (mL^.^kg^.^min^−1^)	49.3		±4.7
Maximum V′E (L^.^min^−1^)	125.9		±12.4
Maximal heart rate (b^.^min^−1^)	178		±8.9
Maximum RER	1.20		±0.1
Test duration (min:s)	12.5		±1.6
**30** **min exercise intervention data**			
Average % of O_2_ peak during30 min run	80.8	±7.1	
Average V′E (L^.^min^−1^)	94.6	±14.3	
Average heart rate (b^.^min^−1^)	163.7	±12.9	
Average RER	1.0	±0.1	

O_2_ peak (highest oxygen consumption achieved in test); V′E (minute ventilation); RER (respiratory exchange ratio).

### Lymphocyte Response to Exercise

**Figure 2:**
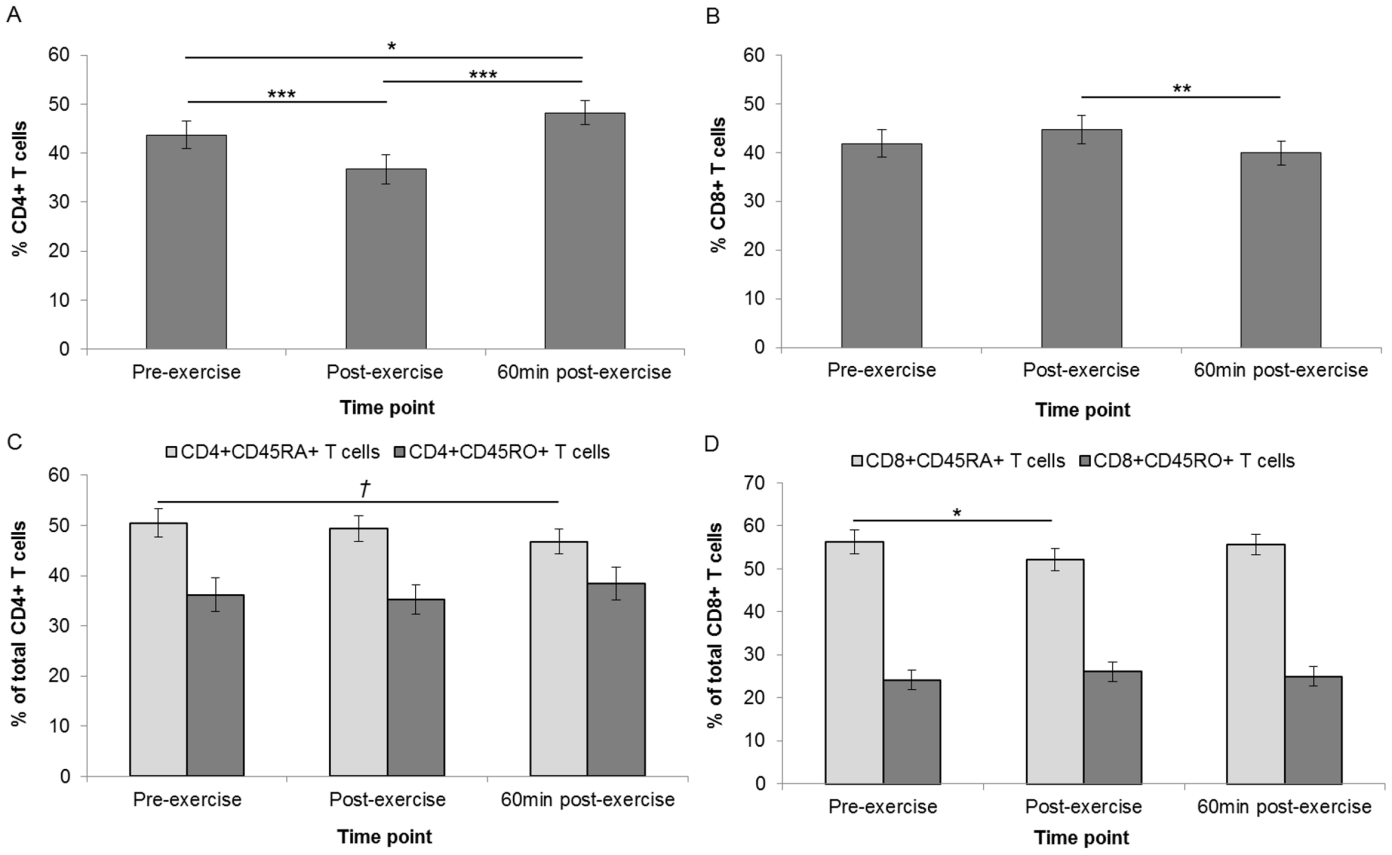
Exercise-induced changes in T cell populations. T cell populations were measured at each time point and expressed as a relative percentage of CD3+ T cells (n = 22) in both CD4+ T cells (**A**) and CD8+ T cells (**B**). Relative changes in CD45RA+ and CD45RO+ phenotypes were assessed in CD4+ T cells (**C**) and CD8+ T cells (**D**) respectively. Error bars indicate SEM. *†* indicates *P = *0.05, *indicates *P*<0.05, ***indicates *P*<0.001.

Relative frequencies of CD4+ T cells (expressed as a percentage of CD3+ T cells) decreased from 43.7% pre-exercise to 36.7% post-exercise (*P*<0.001) and increased to 48.2% 60 min post-exercise (*P*<0.001) (Figure 2). The relative percentage of CD8+ T cells (expressed as a percentage of CD3+ T cells) underwent a non-significant increase from 41.9% pre-exercise to 44.8% post-exercise before significantly decreasing to 39.9% 60 min post-exercise (*P*<0.01). The relative frequency of CD4+CD45RA+ T cells (expressed as a percentage of CD4+ T cells) decreased from 49.5% post-exercise to 46.8% 60 min post-exercise (*P = *0.05). The relative frequency of CD8+CD45RA+ T cells decreased from 56.3% pre-exercise to 52.1% post-exercise (*P*<0.05) before returning to pre-exercise levels 60 min post-exercise. There were no significant changes in either of the CD45RO+ subsets.

### The Acute Effect of Cardiorespiratory Exercise on Genome Wide miRNA Expression

Fifty-six miRNAs were significantly differentially regulated in ten healthy males after 30 minutes of intense cardiorespiratory exercise (Table S5).

### qPCR Validations of Selected miRNAs

**Figure 3:**
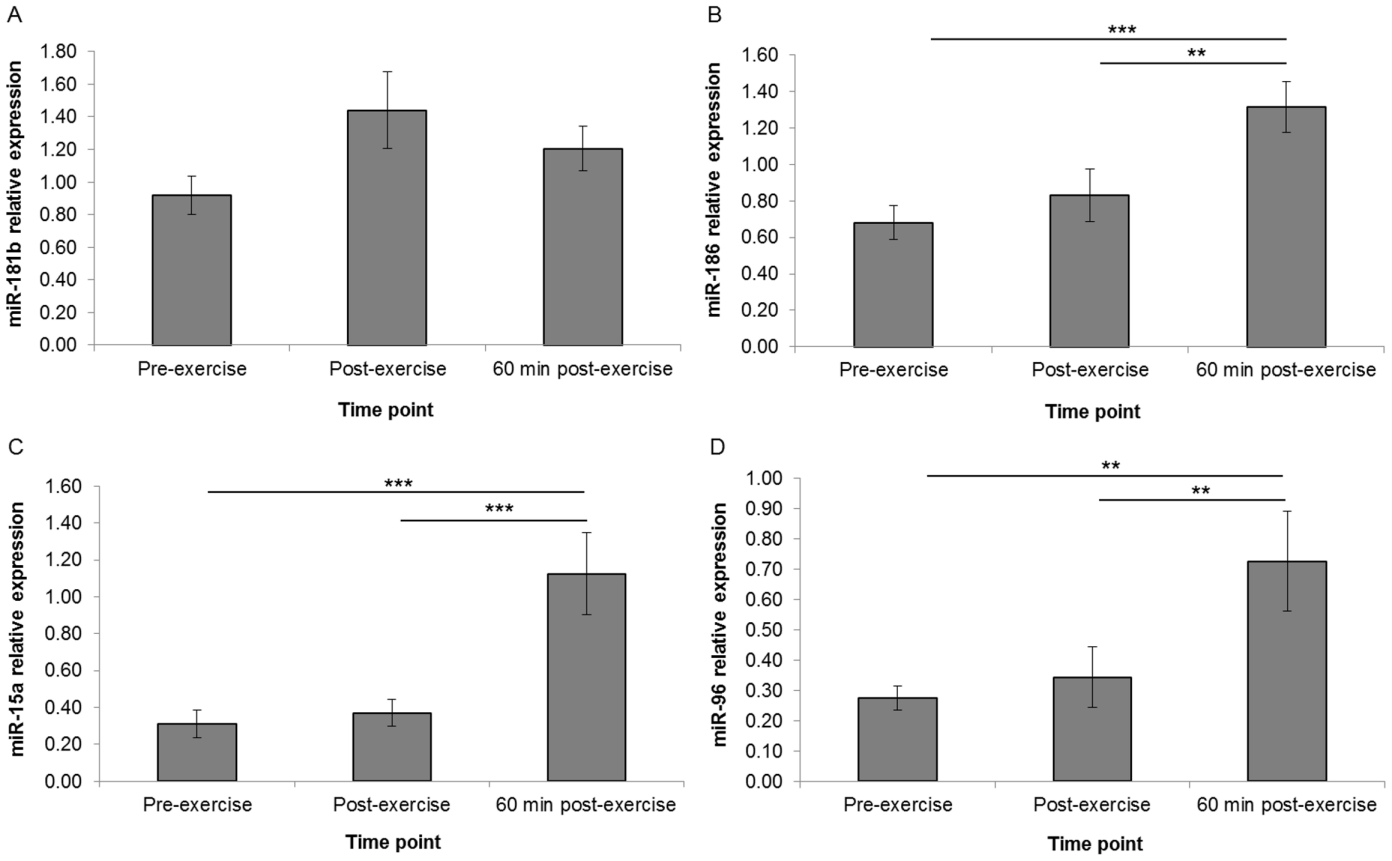
Differential regulation of selected miRNAs in unsorted WBCs. Relative expression of each target miRNA was assessed at pre-exercise, post-exercise and 60 minutes post-exercise (n = 18). Whilst only a strong trend was observed for miR-181b (**A**), significant changes in regulation were observed for miR-186 (**B**), miR-15a (**C**), and miR-96 (**D**). All data is expressed relative to an average of RNU44 and RNU48. Error bars indicate SEM. *indicates *P*<0.05 and **indicates *P*<0.01, and ***indicates *P*<0.001.

The qPCR validations (n = 18) identified a non-significant, post-exercise upregulation trend in miR-15a (fold change = 1.25), miR-181b (fold change = 1.49), miR-186 (fold change = 1.18) and miR-96 (fold change = 1.14) (Figure 3). Statistically significant upregulation between pre- and 60 min post-exercise was observed in miR-186 (fold change = 1.93, *P<0.001*), miR-15a (fold change = 3.61, *P*<0.001) and miR-96 (fold change = 2.63, *P*<0.001) (Figure 3). Statistically significant upregulation also occurred between post- and 60 min post-exercise in miR-186 (fold change = 1.58, *P<0.01*), miR-15a (fold change = 3.04, *P*<0.001), and miR-96 (fold change = 2.11, *P*<0.01).

### The Effect of Exercise on T Cell Subset miRNA Expression

**Figure 4:**
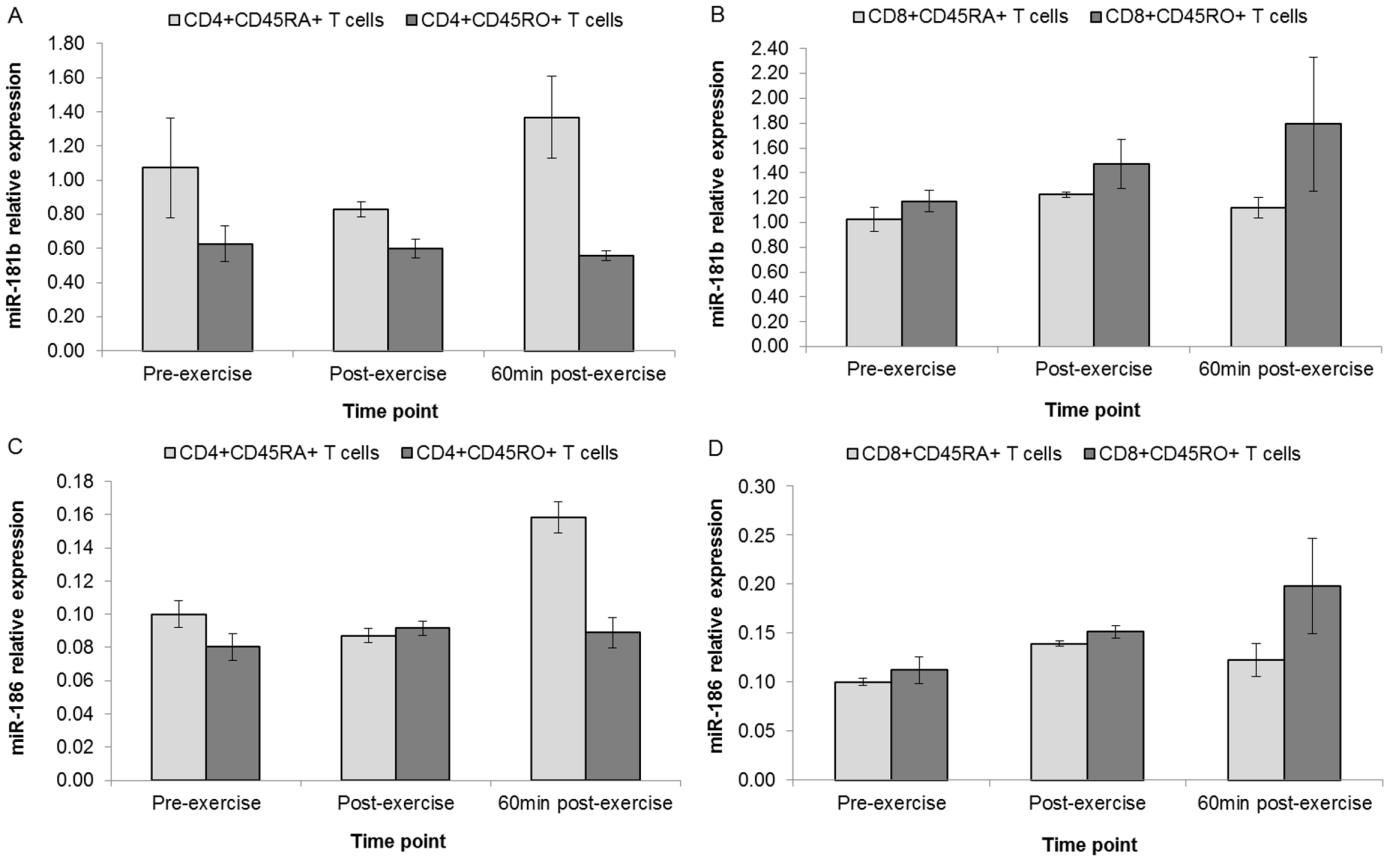
Differential regulation of selected miRNAs in sorted T cell subset pools. (n = 22): Each miRNA was assessed in T cell subset pools. miR-181b was expressed in CD4+CD45RA+ and CD4+CD45RO+ T cells (**A**) and CD8+CD45RA+ and CD8+CD45RO+ T cells (**B**). miR-186 was also expressed in CD4+CD45RA+ and CD45RO+ T cells (**C**) and CD8+CD45RA+ and CD8+CD45RO+ T cells (**D**).

Only miR-181b and miR-186 were detected in the sorted T cell subsets. The expression profile of miR-181b demonstrated a biphasic post-exercise downregulation in CD4+CD45RA+ and a marginal post-exercise increase in CD8+CD45RA+ T cells (Figure 4). There was no appreciable regulation in miR-181b for CD4+CD45RO+ T cells whilst CD8+CD45RO+ T cells exhibited a stepwise increase. Expression of miR-186 exhibited a 60 min post-exercise increase in CD4+CD45RA+ T cells and CD8+CD45RO+ T cells showed a post-exercise stepwise increase.

### Telomere Gene Pathway Expression in Pooled Samples

Fold changes in relative expression appear in Table S6. The TaqMan Human Telomere Extension array was only performed once due to limited sample; therefore results are limited to fold change without corresponding measures of statistical significance. In response to acute exercise, 16 of the 28 telomeric genes were upregulated from pre- to post-exercise and 15 were upregulated from pre- to 60 min post-exercise. A total of 10 genes were marginally downregulated between pre-post exercise and 13 were downregulated from pre- to 60 min post-exercise.

### Validation of Gene Expression in Individual Subjects

**Figure 5:**
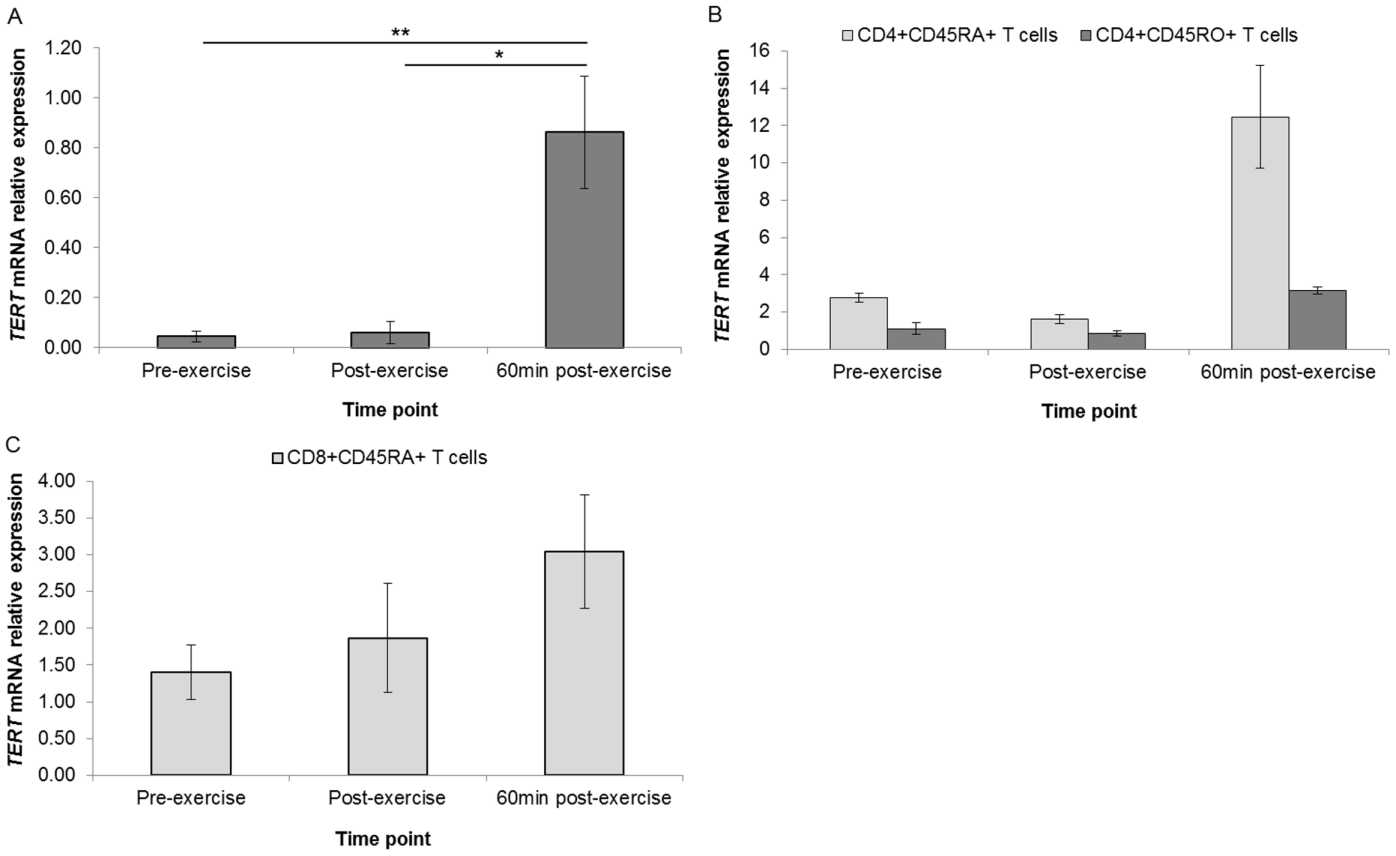
Differential regulation of *TERT* mRNA expression. *TERT* mRNA expression was assessed in unsorted WBCs (n = 17) (**A**), CD4+CD45RA+ and CD4+CD45RO+ T cells (pool of n = 22) (**B**), and CD8+CD45RA+ T cells (pool of n = 22) (**C**). *TERT* was not detectable in CD8+CD45RO+ T cells. Gene expression data is expressed relative to endogenous reference gene (*GAPDH*). *indicates *P*<0.05, **indicates *P*<0.01.

We further validated expression levels of *TERT* mRNA by qPCR based on the fold change and the important role it plays in telomere homeostasis. We also investigated the expression of *SIRT6* mRNA (not featured on the TaqMan Human Telomere Extension array) as this gene plays a role in telomeric chromatin maintenance [37]. Despite low basal expression levels, WBC *TERT* mRNA expression exhibited a significant increase from pre- to 60 min post-exercise (fold change = 19.4, *P = *0.001) (Figure 5). To determine if a particular T cell subset was driving the observed changes, population specific pools were assessed over the three time points. The stepwise upregulation trend was broadly confirmed in CD4+CD45RA+, CD4+CD45RO+ and CD8+CD45RA+ T cell subsets (Figure 5).

**Figure 6:**
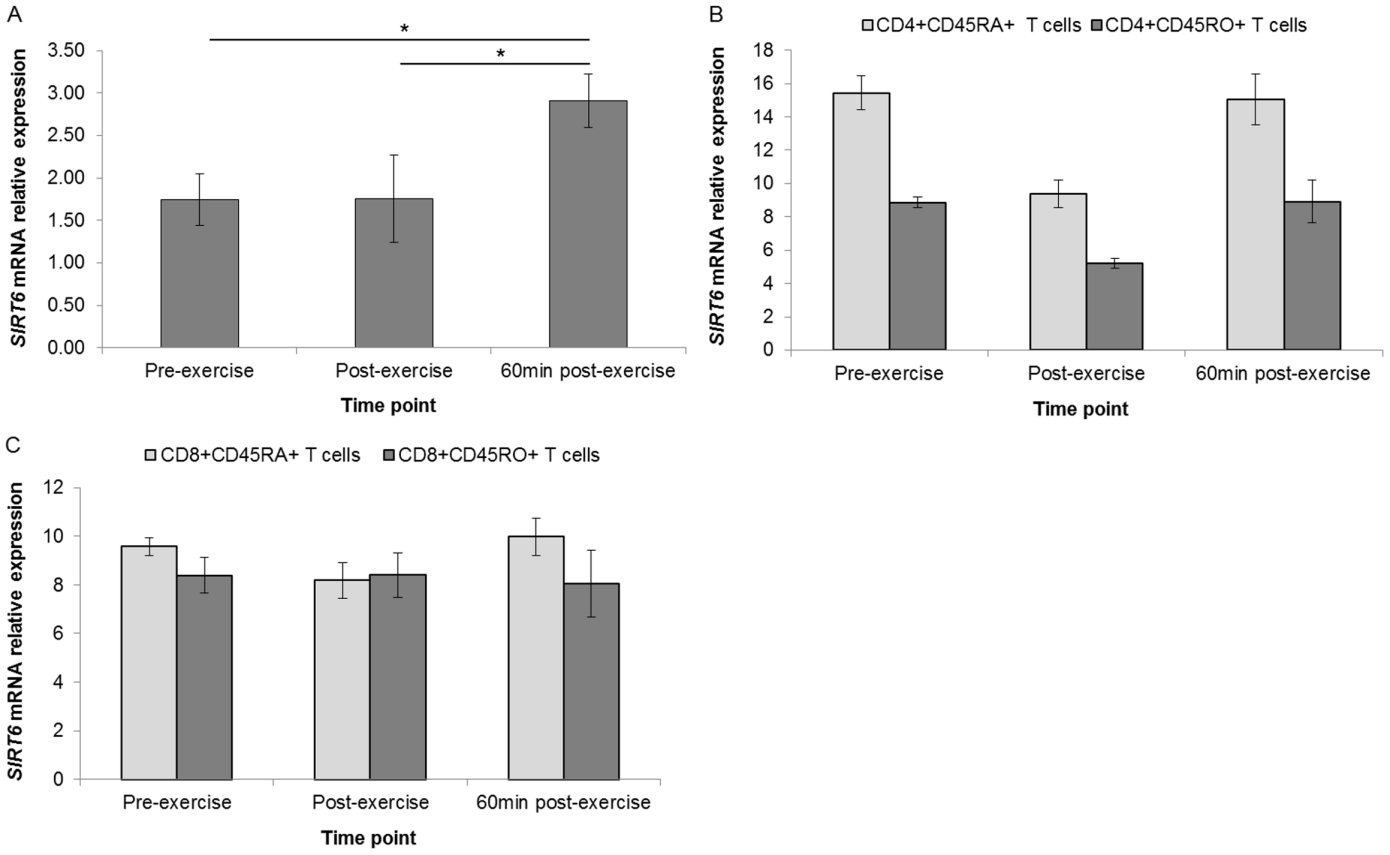
Differential regulation of *SIRT6* mRNA expression. In Unsorted WBCs (n = 17) (**A**), CD4+CD45RA+ and CD4+CD45RO+ T cells (pool of n = 22) (**B**), and CD8+CD45RA+ and CD8+CD45RO+ T cells (pool of n = 22) (**C**). Gene expression data is expressed relative to endogenous reference gene (*GAPDH*). *indicates *P*<0.05.

*SIRT6* mRNA expression showed significant upregulation in WBCs between pre- and 60 min post-exercise (fold change = 1.67, *P*<0.05) and between post- and 60 min post-exercise (fold change = 1.66, *P*<0.05). *SIRT6* was downregulated immediately post-exercise in CD4+CD45RA+, CD8+CD45RA+ and CD4+CD45RO+ T cell subset pools (Figure 6).

### miRNA Target Gene Prediction

We used *miRGen Targets* prediction software to determine potential target gene transcripts of the selected miRNAs. We chose to validate the expression profiles of the potential miRNA target gene transcripts for *TERF2IP* and *RAD50* using qPCR. The pre- to 60 min post-exercise upregulation of the selected miRNAs did not result in statistically significant downregulation of the transcript targets over the same time period. The downregulation of miR-181b expression between post- and 60 min post-exercise showed marginal significance (fold change = 0.84, *P = *0.05) and paralleled significant upregulation of potential target *TERT* transcript (fold change = 14.75, *P*<0.05). Expression increases in miR-186 from post- to 60 min post-exercise (fold change = 1.58, *P*<0.001) paralleled a significant upregulation of *SIRT6* mRNA (fold change = 1.66, *P*<0.05), a significant downregulation of *TERF2IP* mRNA (fold change = 0.54, *P*<0.01) and a strong trend towards significance for *RAD50* (fold change = 0.76, *P = *0.05). Expression increases in miR-96 from post- to 60 min post-exercise (fold change = 2.11, *P*<0.01) also paralleled simultaneous downregulation in *TERF2IP* mRNA expression (fold change = 0.54, *P*<0.01) (Table S7).

**Figure 7:**
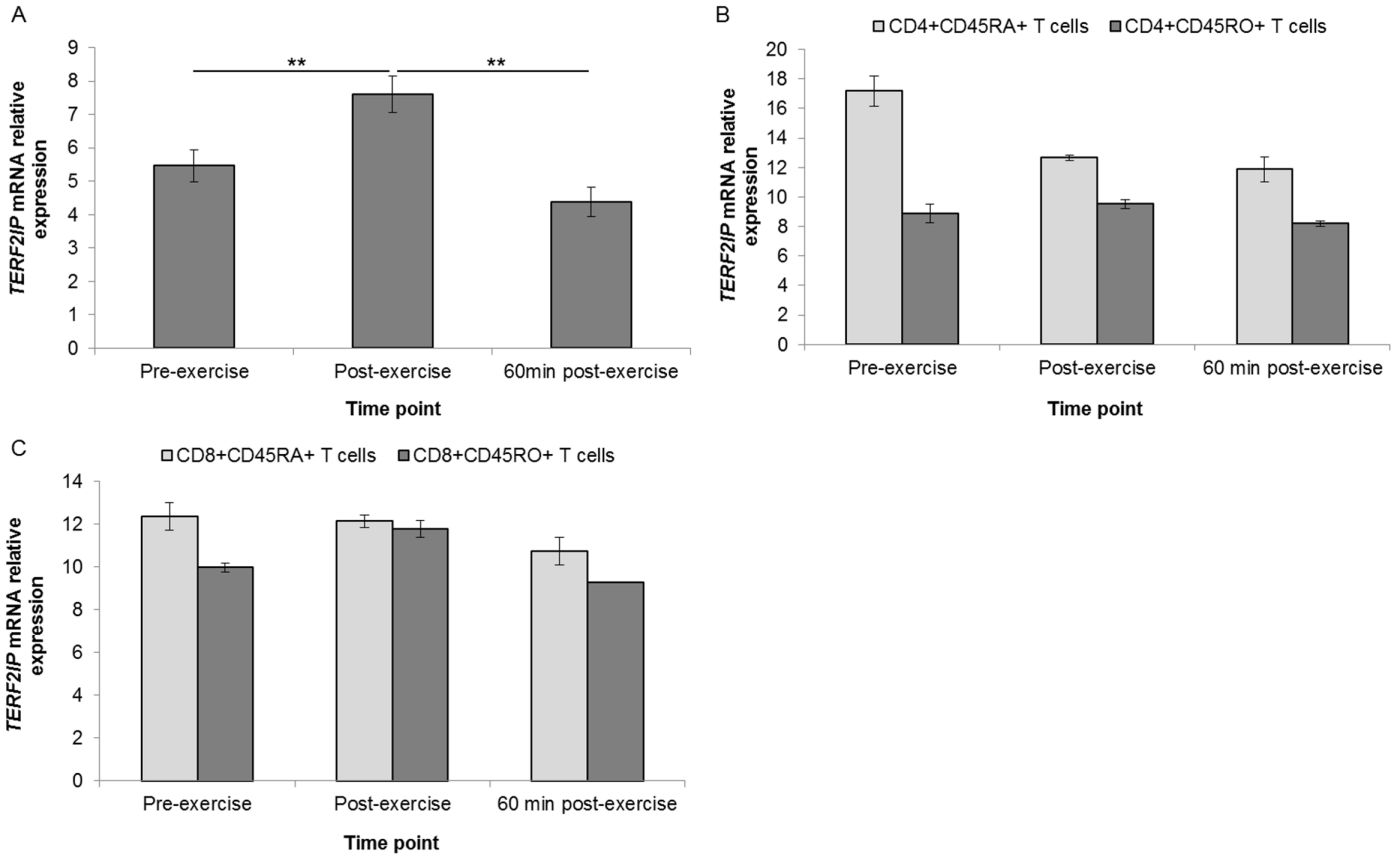
Differential regulation of *TERF2IP* mRNA interactions. Relative expression in WBCs (n = 16) (**A**), in CD4+CD45RA+ and CD4+CD45RO+ T cell pools (n = 22) (**B**), and in CD8+CD45RA+ and CD8+CD45RO+ T cells pools (n = 22) (**C**). Gene expression data is expressed relative to endogenous reference gene (*GAPDH*). **indicates *P*<0.01.

**Figure 8:**
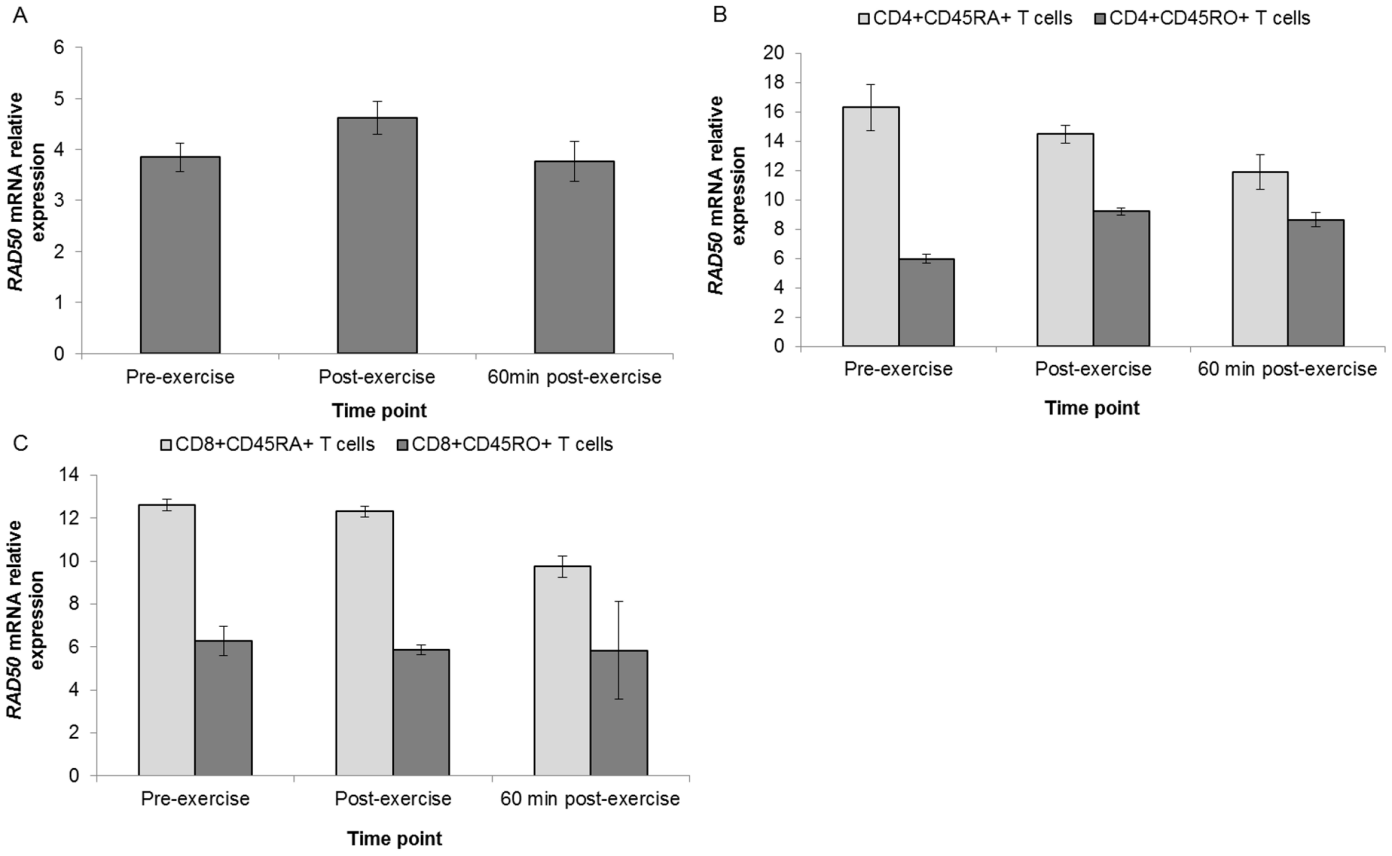
Differential regulation of *RAD50* mRNA. Relative expression in WBCs (n = 16) (**A**), in CD4+CD45RA+ and CD4+CD45RO+ T cell pools (n = 22) (**B**), and in CD8+CD45RA+ and CD8+CD45RO+ T cells pools relative expression (n = 22) (**C**). Gene expression data is expressed relative to endogenous reference gene (*GAPDH*).

White blood cell expression of *TERF2IP* mRNA was upregulated immediately post exercise (fold change = 1.46, *P*<0.01); but showed significant downregulation from post- to 60 min post-exercise (fold change = 0.54, *P*<0.01) (Figure 7). *TERF2IP* mRNA expression demonstrated a stepwise downregulation from pre- to 60 min post-exercise in CD4+CD45RA+ and CD8+CD45RA+ T cell subset pools. *RAD50* exhibited a non-significant post-exercise upregulation of mRNA expression in WBCs followed by a return to resting levels (*P = *0.28). A stepwise downregulation from pre- to 60 min post-exercise was observed in CD4+CD45RA+ and CD8+CD45RA+ T cell subset pools (Figure 8).

## Discussion

Here we report for the first time that acute exercise can lead to the transcriptional regulation of several key telomeric genes in immune cells. First, we report the upregulation of *TERT* mRNA which plays a critical molecular role in telomere maintenance [38]. Second, we report that exercise regulates miRNAs with the potential to control the downstream expression of genes involved in telomere homeostasis. The exercise-induced regulation of telomeric genes and miRNAs may provide an important mechanistic link between physical activity, telomeres and improved health. This is an important finding given the extensive evidence linking accelerated telomere shortening to several chronic diseases [39], many of which can be ameliorated by aerobic exercise [40]. Telomere homeostasis underpins the function of several immune cell subsets [41], which in turn play critical roles in chronic pathology such as age-related diseases [42], atherosclerosis [43] and metabolic diseases [44].

Exercise-induced miRNA regulation exhibits exquisite specificity for both tissue type and exercise modality. Differential expression profiles have been identified between muscle contraction types [45], aerobic fitness levels [23] and resistance training adaptability [46]. Recent studies have assessed miRNA expression in leukocyte subsets, identifying differential regulation of 38 miRNAs in neutrophils [22], 23 miRNAs in natural killer cells [35] and 34 miRNAs in peripheral blood mononucleated cells (PBMCs) [21], [22], [35]. Thirty eight miRNAs were differentially expressed in neutrophils after ten 2 minute bouts of intense cycle ergometry [22]. Pathway analysis of miRNA targeted genes identified pathways responsible for neutrophil immune function and apoptosis [47], [48], chronic inflammation [49] and immune function [50]. The analysis of PBMC miRNA revealed 34 significantly regulated miRNAs that influenced genes associated with 12 signalling pathways including pro- and anti-inflammatory cytokine regulation [51]–[53], lymphocyte activation and differentiation [54], cell communication [55] and cancer [56].

The current study provides a unique and unprecedented snap shot of over 1300 miRNAs, revealing 56 significantly regulated in response to exercise. *In silico* analyses predicted miRNA/mRNA interactions between the following miRNAs and telomeric gene transcripts: miR-181b and *TERT*, miR186 and *TERF2IP, RAD50* and *SIRT6*, miR-96 and *TERF2IP* and miR-15a and TATA box binding protein (*TBP*). Validations conducted for *TERT* and *SIRT6* showed significant 60 min post-exercise increases that paralleled concomitant increases in the potential binding miRNA, effectively dismissing any significant miRNA/transcript interaction. Validations for *TERF2IP* and *RAD50* revealed significantly decreased transcript abundance at 60 min post-exercise for *TERF2IP* only, paralleled by concomitant increases in potential binding miRNAs (miR-186 and miR-96).

Two miRNAs investigated in the present study; miR-181b and miR-96, have previously been identified in neutrophils following 30 minutes of exercise [22]. Expression profiles of miR-181b were broadly confirmed at the same time point between the two studies; however, miR-96 was downregulated in the previous study and upregulated in the present study. This may be due to analysis of whole WBCs in the present study (including neutrophils) as opposed to isolated neutrophils in the previous study. Pathway analysis identified 578 genes targeted by miR-96 and 652 genes targeted by miR-181b [22]. A more recent study identified upregulation of miR-181b and miR-15a in PBMCs immediately after 30 minutes of intermittent aerobic exercise [21]. Both miR-181b and miR-15a were identified in the present study; however, only miR-15a was significantly regulated. Utilizing a similar exercise protocol, a recent study identified four miRNA-mRNA networks dynamically regulated by 30 minutes of exercise [57]. The target mRNAs are involved in apoptosis, immune function, transcription regulation and membrane traffic of proteins.

RAD50 associates with meiotic recombination 11 (MRE11) and nibrin (NBS1) to form the MRN complex which repairs DNA damage and assists telomere structure [58], [59]. The MRN complex also positively regulates telomerase-dependent telomere elongation via an interaction with telomeric repeat binding factor 1 (TERF1) and ataxia telangiectasia mutated (ATM) [60]. The complex formed by RAD50 and MRE11 is thought to help stabilize t-loop formation [61]. There is no immediately intuitive reason for the post-exercise downward trend in *RAD50* mRNA expression. Possible hypotheses include transiently compromised stability of the shelterin complex and suppression of DNA damage machinery at the telomere by the heavily fortified shelterin complex [62]. Telomeric DNA is preferentially damaged by oxidative stress [63]; however, the conformation of the shelterin complex and T-loop may preclude access to sites of DNA damage. Mass spectrometry and affinity purification have also identified an association between RAD50 and the TERF2IP*/*telomere repeat binding factor 2 (TRF2) protein complex [64]. This association suggests that *RAD50* mRNA expression may parallel that of *TERF2IP* mRNA.

The hypothesis of exercise-induced shelterin instability is reinforced by the simultaneous decrease in *TERF2IP* mRNA, the protein of which forms part of the shelterin complex and is recruited to telomeres via interaction with *TRF2* (also known as *TERF2*) [65]. TERF2IP deficiency reduces telomere stability and increases recombination [66]. Equivocal results have identified TERF2IP as both a negative regulator [64] and a positive regulator of telomere length [65]. Additional roles for TERF2IP include prevention of non-homologous end joining [67] and homology-directed repair [66], protection from obesity via regulation of metabolic genes [68] and regulation of senescence [69]. Additionally, TERF2IP associates with iκB kinases in the cytoplasm and regulates NF-κB modulated gene expression [70].

To our knowledge, this study is the first to report the upregulation of *TERT* mRNA after acute aerobic exercise in WBCs and T cell subsets. The current findings may provide a potential mechanistic link between physical activity and telomere length. Transcriptional plasticity of *TERT* is important for the adaptive immune system; the replicative capacity of which depends upon the telomere length and TERT expression of the constituent T cells [71]. Ectopic expression of *TERT* has been shown to increase CD4+ and CD8+ proliferative capacity and enhance resistance to oxidative stress and apoptosis [72]–[74]. Unlike T cells, mature granulocytes of the innate immune system do not undergo successive rounds of division therefore granulocyte telomere length is a function of myeloid progenitor cells [75]. Accordingly, telomerase activity within the granulocyte population is low [76] to undetectable [77], [78]. Whilst the WBC fraction analysed in the present study contained elements of both the adaptive and innate immune system, all findings were subsequently validated in sorted populations of T cells subsets.

Epel et al., (2010) identified an 18% increase in leukocyte telomerase within one hour of an acute psychological stressor [79]. The increase was independent of immune cell redistribution and was positively associated with concomitant increases in cortisol. The authors postulated a possible protective function for the acute increase in telomerase, such as preparation for immune cell proliferation [79]. The authors also indicated that phosphorylation of telomerase and/or changes in subcellular localization likely account for the acute increases in telomerase, given the extended timeline likely required for transcriptional regulation and/or alternate splicing of *TERT* [79]. The findings of the present study are in opposition to this hypothesis, demonstrating transcriptional regulation of *TERT* within 60 minutes of exercise.

There is significant evidence of a linear relationship between *TERT* mRNA expression and telomerase activity in a range of tissues [80]–[84]. Despite this, numerous human tissues exhibit significant heterogeneity between the two, indicating potential post-transcriptional modification of *TERT*. Gizard et al., (2011) found that macrophages stimulated with lipopolysaccharide, oxidized low density lipoprotein and tumour necrosis factor-α exhibited induction of *TERT* mRNA expression that paralleled inducible telomerase activity [80]. However, macrophages stimulated with interleukin-1β exhibited only modest increases in *TERT* mRNA despite high induction of telomerase activity [80]. Although demonstrating an overall correlation between *TERT* mRNA and telomerase activity in lung cancer tissues, Hara et al., (2001) reported that 12.9% of samples exhibited *TERT* mRNA without telomerase activity and an additional 12.9% demonstrated telomerase activity without detectable *TERT* mRNA expression [81].

Human thymocytes, tonsil and peripheral blood T and B cells have all been shown to exhibit *TERT* mRNA expression independent of telomerase activity [85]. All lymphocyte subsets harvested from peripheral blood and thymus contain *TERT* mRNA and protein irrespective of telomerase activity [86]. Significant discrepancies between *TERT* mRNA expression and telomerase activity have also been identified in soft tissue sarcomas [87] and non-small cell lung cancer [88]. Additionally, expression levels of *TERT* mRNA do not regulate telomerase activity in telomerase-reconstituted primary human fibroblast clones [89].

The numerous associations between exercise, telomere length and telomerase are observational in design with either confounding lifestyle factors or discrepant blood collection timelines. A recent study identified no change in PBMC *TERT* mRNA expression or telomerase levels after 7 days of ultra-marathon running in trained athletes [90]. Given that measurement occurred the day after marathon completion, acute changes in gene expression or T cell frequencies may have regressed to basal levels within this time frame. The current study is unique in that it used an acute measurement timeline; providing a snapshot of transcriptional changes immediately after and 60 min after exercise. Parallel changes in cell population frequencies were also assessed to account for subset redistribution.

The acute inflammatory milieu induced by exercise may in part be responsible for signalling increases in WBC *TERT* mRNA. Previous research has shown pro-inflammatory signalling induces *TERT* mRNA expression and telomerase activity in macrophages [80]. Intense exercise transiently and preferentially redistributes T cell subsets [91], [92]. Studies have equivocally identified preferential mobilization of T cells with shortened telomeres [93], senescent phenotypes with longer telomeres [94] and CD45RA+ effector-memory phenotypes [95]. We demonstrated a non-significant 60 min post-exercise decline in CD4+CD45RA+ T cells and a non-significant increase in CD4+CD45RO+ T cell frequency. Telomere length was not measured in the present study because the focus was acute regulation of telomeric genes. Given the average trajectory of leukocyte telomere shortening [96]–[98], genuine changes in telomere length would not be detectable after 60 minutes. Additionally, telomere length appears sensitive to the stage of T cell differentiation [94]; a factor our flow cytometry staining panel could not accurately discriminate.

Exhaustive exercise has been shown to increase human PBMC expression of *SIRT1* and decrease *SIRT3* and *SIRT4* mRNA [99]. To our knowledge, this is the first study to characterize exercise-induced *SIRT6* mRNA expression in humans. *SIRT6* is a chromatin binding protein existing predominantly in the nucleus [100]. SIRT6 influences telomeric chromatin by deacetylating H3K9 and H3K56 [37], thereby reducing chromatin accessibility. SIRT6 also plays an important role in DNA repair mechanisms by modulating base excision repair (BER) [101] and double strand break (DSB) repair [102]. SIRT6 is recruited to DSB sites under conditions of oxidative stress [103]. Other extra-telomeric roles for SIRT6 include enhanced mitochondrial respiration [104], transcriptional regulation of gene expression [105], positive regulation of pro-inflammatory cytokines [106] and systemic glucose metabolism [107]. The post-exercise upregulation of *SIRT6* mRNA exhibited in unsorted WBCs was not replicated in sorted T cell populations. A likely explanation for the disparate results is that the predominant cell type(s) driving the expression changes were not T cells and were therefore excluded when T cells were positively selected.

There are some inherent limitations to this study. Without *a priori* knowledge of the precise time course of miRNA expression, it is possible that the time course used in this study may have missed the greatest magnitude of change. Additionally, the magnitude of miRNA expression needed to elicit detectable decreases in target mRNA is unknown. Determining the optimal timeframe in which to measure telomeric gene expression also presents a challenge as little is known about the exercise-induced transcriptional timeline or half-life of telomeric gene transcripts. This study assessed changes in mRNA expression and did not quantify protein or functional enzyme status. Additionally, we were not able to differentiate between the telomeric and extra-telomeric roles of the genes and miRNAs in this study. Observed increases in *SIRT6* and *TERT* mRNAs may have resulted from the upregulation of extra-telomeric pathways. Additional reporter assay and gain/loss of function experiments are needed to confirm the legitimacy of the miRNA/mRNA interactions.

The low sample yield from sorted T cell populations necessitated pooling into cell/time point specific pools for analysis. Whilst this provided interesting, subpopulation-wide overviews, it precluded additional individual validations and robust statistical analysis. Whilst the analysis was predominantly conducted in WBCs and T cell subsets, analysis of other leukocyte subsets such as B cells and neutrophils may help form a more complete picture.

Cytomegalovirus (CMV) is a persistent and ubiquitous herpes virus that can affect T cell telomere/telomerase homeostasis [108]–[110]. The CMV seroprevalance rate in Australian males aged 20–24 years is 50% [111], with global seroprevalence rates exceeding 70% above 60 years of age [112]. Financial and logistical constraints precluded the individual testing for CMV seropositivity and we felt that testing and excluding positive individuals would place further constraints on an already small sample size. Potential participants were excluded if they had been diagnosed with glandular fever, chronic fatigue syndrome or other viral infections lasting longer than 3 months. Given the asymptomatic nature of CMV infection, we are unable to exclude the possibility that some participants were unknowingly seropositive.

In conclusion we have shown that 30 minutes of cardiorespiratory exercise is sufficient to elicit an upregulation of key telomeric gene *TERT* mRNA and the downregulation of *TERF2IP* mRNA. We also showed the differential regulation of 56 miRNAs, including miR-186 and miR-96 which have potential transcriptional influence on telomeric gene transcripts. These results may provide a mechanistic insight into pathways via which exercise of appropriate intensity may mediate improved telomere homeostasis and physical health.

## Supporting Information

Table S1 **Selected miRNAs and their potential mRNA interactions.**
(DOCX)

Table S2 **Quantitative real-time PCR TaqMan microRNA assays.**
(DOCX)

Table S3 **Quantitative real-time PCR gene expression primers (SYBR Green chemistry) and conditions.**
(DOCX)

Table S4 **Quantitative real-time PCR TaqMan gene expression assays.**
(DOCX)

Table S5 **Significantly regulated miRNAs detected in genome-wide microarray.**
(DOCX)

Table S6 **Differential regulation obtained using the TaqMan Telomere extension array.**
(DOCX)

Table S7 **Parallel regulation of miRNAs and corresponding potential target transcripts.**
(DOCX)

## References

[1] Cherkas LF, Hunkin JL, Kato BS, Richards JB, Gardner JP, et al (2008) The association between physical activity in leisure time and leukocyte telomere length. Arch Intern Med 168: 154–158 10.1001/archinternmed.2007.39 18227361

[2] Denham J, Nelson CP, O’Brien BJ, Nankervis SA, Denniff M, et al (2013) Longer leukocyte telomeres are associated with ultra-endurance exercise independent of cardiovascular risk factors. PLOS ONE 8: e69377 10.1371/journal.pone.0069377 23936000 PMC3729964

[3] LaRocca TJ, Seals DR, Pierce GL (2010) Leukocyte telomere length is preserved with aging in endurance exercise-trained adults and related to maximal aerobic capacity. Mechanisms of ageing and development 131: 165–167. 20064545 10.1016/j.mad.2009.12.009 PMC2845985

[4] Ludlow AT, Zimmerman JOB, Witkowski S, Hearn JOEW, Hatfield BD, et al (2008) Relationship between physical activity level, telomere length, and telomerase activity. Med Sci Sports Exerc 40: 1764–1771 10.1249/MSS.0b013e31817c92aa 18799986 PMC2581416

[5] Puterman E, Lin J, Blackburn E, O’Donovan A, Adler N, et al (2010) The power of exercise: Buffering the effect of chronic stress on telomere length. PLoS One 5: e10837 10.1371/journal.pone.0010837 20520771 PMC2877102

[6] Werner C, Furster T, Widmann T, Poss J, Roggia C, et al (2009) Physical exercise prevents cellular senescence in circulating leukocytes and in the vessel wall. Circulation 120: 2438–2447 10.1161/CIRCULATIONAHA.109.861005 19948976

[7] Aviv A, Valdes A, Gardner JP, Swaminathan R, Kimura M, et al (2006) Menopause modifies the association of leukocyte telomere length with insulin resistance and inflammation. J Clin Endocrinol Metab 91: 635–640 10.1210/jc.2005-1814 16303830

[8] Benetos A, Okuda K, Lajemi M, Kimura M, Thomas F, et al (2001) Telomere length as an indicator of biological aging: The gender effect and relation with pulse pressure and pulse wave velocity. Hypertension 37: 381–385 10.1161/01.HYP.37.2.381 11230304

[9] Brouilette S, Singh RK, Thompson JR, Goodall AH, Samani NJ (2003) White cell telomere length and risk of premature myocardial infarction. Arterioscler Thromb Vasc Biol 23: 842–846 10.1161/01.ATV.0000067426.96344.32 12649083

[10] Gardner JP, Li S, Srinivasan SR, Chen W, Kimura M, et al (2005) Rise in insulin resistance is associated with escalated telomere attrition. Circulation 111: 2171–2177 10.1161/01.CIR.0000163550.70487.0B 15851602

[11] Jeanclos E, Schork NJ, Kyvik KO, Kimura M, Skurnick JH, et al (2000) Telomere length inversely correlates with pulse pressure and is highly familial. Hypertension 36: 195–200 10.1161/01.HYP.36.2.195 10948077

[12] Nawrot TS, Staessen JA, Gardner JP, Aviv PA (2004) Telomere length and possible link to X chromosome. The Lancet 363: 507–510 10.1016/S0140-6736(04)15535.9 14975611

[13] Samani NJ, Boultby R, Butler R, Thompson JR, Goodall AH (2001) Telomere shortening in atherosclerosis. The Lancet 358: 472–473 10.1016/S0140-6736(01)05633-1 11513915

[14] Sampson MJ, Winterbone MS, Hughes JC, Dozio N, Hughes DA (2006) Monocyte telomere shortening and oxidative DNA damage in type 2 diabetes. Diabetes Care 29: 283–289 10.2337/diacare.29.02.06.dc05-1715 16443874

[15] Valdes AM, Andrew T, Gardner JP, Kimura M, Oelsner E, et al (2005) Obesity, cigarette smoking, and telomere length in women. The Lancet 366: 662–664 10.1016/S0140-6736(05)66630-5 16112303

[16] de Lange T (2005) Shelterin: The protein complex that shapes and safeguards human telomeres. Genes Dev 19: 2100–2110 10.1101/gad.1346005 16166375

[17] Butt HZ, Atturu G, London NJ, Sayers RD, Bown MJ (2010) Telomere length dynamics in vascular disease: A review. European Journal of Vascular and Endovascular Surgery 40: 17–26 10.1016/j.ejvs.2010.04.012 20547081

[18] Friedrich U, Griese EU, Schwab M, Fritz P, Thon KP, et al (2000) Telomere length in different tissues of elderly patients. Mech Ageing Dev 119: 89–99 10.1016/S0047-6374(00)00173-1 11080530

[19] Okuda K, Bardeguez A, Gardner JP, Rodriguez P, Ganesh V, et al (2002) Telomere length in the newborn. Pediatr Res 52: 377–381 10.1203/00006450-200209000-00012 12193671

[20] Wilson WR, Herbert KE, Mistry Y, Stevens SE, Patel HR, et al (2008) Blood leukocyte telomere DNA content predicts vascular telomere DNA content in humans with and without vascular disease. Eur Heart J 29: 2689–2694 10.1093/eurheartj/ehn386 18762552

[21] Radom-Aizik S, Zaldivar Jr F, Leu SY, Adams GR, Oliver S, et al (2012) Effects of exercise on microRNA expression in young males peripheral blood mononuclear cells. Clinical and Translational Science 5: 32–38 10.1111/j.1752-8062.2011.00384.x 22376254 PMC4664183

[22] Radom-Aizik S, Zaldivar F, Oliver S, Galassetti P, Cooper DM (2010) Evidence for microRNA involvement in exercise-associated neutrophil gene expression changes. Journal of Applied Physiology 109: 252–261 10.1152/japplphysiol.01291.2009 20110541 PMC2904199

[23] Bye A, Røsjø H, Aspenes ST, Condorelli G, Omland T, et al (2013) Circulating MicroRNAs and aerobic Fitness–The HUNT-study. PLOS ONE 8: e57496 10.1371/journal.pone.0057496 23469005 PMC3585333

[24] Baggish AL, Hale A, Weiner RB, Lewis GD, Systrom D, et al (2011) Dynamic regulation of circulating microRNA during acute exhaustive exercise and sustained aerobic exercise training. J Physiol (Lond ) 589: 3983–3994 10.1113/jphysiol.2011.213363 21690193 PMC3179997

[25] García-Cao M, O’Sullivan R, Peters AH, Jenuwein T, Blasco MA (2003) Epigenetic regulation of telomere length in mammalian cells by the Suv39h1 and Suv39h2 histone methyltransferases. Nat Genet 36: 94–99 10.1038/ng1278 14702045

[26] Gonzalo S, García-Cao M, Fraga MF, Schotta G, Peters AH, et al (2005) Role of the RB1 family in stabilizing histone methylation at constitutive heterochromatin. Nat Cell Biol 7: 420–428 10.1038/ncb1235 15750587

[27] Gonzalo S, Jaco I, Fraga MF, Chen T, Li E, et al (2006) DNA methyltransferases control telomere length and telomere recombination in mammalian cells. Nat Cell Biol 8: 416–424 10.1038/ncb1386 16565708

[28] Gigek CO, Leal MF, Silva PN, Lisboa LC, Lima EM, et al (2009) hTERT methylation and expression in gastric cancer. Biomarkers 14: 630–636 10.3109/13547500903225912 20001710

[29] Iliopoulos D, Oikonomou P, Messinis I, Tsezou A (2009) Correlation of promoter hypermethylation in hTERT, DAPK and MGMT genes with cervical oncogenesis progression. Oncol Rep 22: 199–204 10.3892/or00000425 19513524

[30] Wang S, Hu C, Zhu J (2010) Distinct and temporal roles of nucleosomal remodeling and histone deacetylation in the repression of the hTERT gene. Molecular Biology of the Cell 21: 821–832 10.1091/mbc.E09-06-0456 20053684 PMC2828968

[31] Sallusto F, Geginat J, Lanzavecchia A (2004) Central memory and effector memory t cell subsets: Function, generation, and maintenance. Annu Rev Immunol 22: 745–763 10.1146/annurev.immunol.22.012703.104702 15032595

[32] Marques FZ, Campain AE, Tomaszewski M, Zukowska-Szczechowska E, Yang YHJ, et al (2011) Gene expression profiling reveals renin mRNA overexpression in human hypertensive kidneys and a role for MicroRNAs. Hypertension 58: 1093–1098 10.1161/HYPERTENSIONAHA.111.180729 22042811

[33] Brazma A, Hingamp P, Quackenbush J, Sherlock G, Spellman P, et al (2001) Minimum information about a microarray experiment (MIAME)–toward standards for microarray data. Nat Genet 29: 365–371 10.1038/ng1201-365 11726920

[34] Megraw M, Sethupathy P, Corda B, Hatzigeorgiou AG (2007) miRGen: A database for the study of animal microRNA genomic organization and function. Nucleic Acids Research 35: D149–D155 10.1093/nar/gkl904 17108354 PMC1669779

[35] Radom-Aizik S, Zaldivar F, Haddad F, Cooper DM (2013) Impact of brief exercise on peripheral blood NK cell gene and microRNA expression in young adults. Journal of Applied Physiology 114: 628–636. 23288554 10.1152/japplphysiol.01341.2012 PMC3615590

[36] Livak KJ, Schmittgen TD (2001) Analysis of relative gene expression data using real-time quantitative PCR and the 2^−ΔΔCT^ method. Methods 25: 402–408 doi 10.1006/meth.2001.1262. 11846609

[37] Michishita E, McCord RA, Berber E, Kioi M, Padilla-Nash H, et al (2008) SIRT6 is a histone H3 lysine 9 deacetylase that modulates telomeric chromatin. Nature 452: 492–496. 18337721 10.1038/nature06736 PMC2646112

[38] Codd V, Nelson CP, Albrecht E, Mangino M, Deelen J, et al (2013) Identification of seven loci affecting mean telomere length and their association with disease. Nat Genet 45: 422–427 10.1038/ng.2528 23535734 PMC4006270

[39] Calado RT, Young NS (2009) Telomere diseases. N Engl J Med 361: 2353–2365. 20007561 10.1056/NEJMra0903373 PMC3401586

[40] Lee IM, Shiroma EJ, Lobelo F, Puska P, Blair SN, et al (2012) Effect of physical inactivity on major non-communicable diseases worldwide: An analysis of burden of disease and life expectancy. The Lancet 380: 219–229 doi 10.1016/S0140-6736(12)61031-9. PMC3645500 22818936

[41] Weng N (2008) Telomere and adaptive immunity. Mech Ageing Dev 129: 60–66 10.1016/j.mad.2007.11.005 18199471 PMC2276146

[42] Weiskopf D, Weinberger B, Grubeck-Loebenstein B (2009) The aging of the immune system. Transplant Int 22: 1041–1050 10.1111/j.1432-2277.2009.00927.x 19624493

[43] Hansson GK, Hermansson A (2011) The immune system in atherosclerosis. Nat Immunol 12: 204–212 10.1038/ni.2001 21321594

[44] Osborn O, Olefsky JM (2012) The cellular and signaling networks linking the immune system and metabolism in disease. Nat Med 18: 363–374 10.1038/nm.2627 22395709

[45] Banzet S, Chennaoui M, Girard O, Racinais S, Drogou C, et al (2013) Changes in circulating microRNAs levels with exercise modality. Journal of Applied Physiology 115: 1237–1244 10.1152/japplphysiol.00075.2013 23950168

[46] Davidsen PK, Gallagher IJ, Hartman JW, Tarnopolsky MA, Dela F, et al (2011) High responders to resistance exercise training demonstrate differential regulation of skeletal muscle microRNA expression. Journal of Applied Physiology 110: 309–317 10.1152/japplphysiol.00901.2010 21030674

[47] Fortin CF, Larbi A, Dupuis G, Lesur O, Fülöp Jr T (2007) GM-CSF activates the jak/STAT pathway to rescue polymorphonuclear neutrophils from spontaneous apoptosis in young but not elderly individuals. Biogerontology 8: 173–187 10.1007/s10522-006-9067-1 17086367

[48] O’Shea JJ, Murray PJ (2008) Cytokine signaling modules in inflammatory responses. Immunity 28: 477–487 doi 10.1016/j.immuni.2008.03.002. 18400190 PMC2782488

[49] Benson RA, Lowrey JA, Lamb JR, Howie SEM (2004) The notch and sonic hedgehog signalling pathways in immunity. Mol Immunol 41: 715–725 doi 10.1016/j.molimm.2004.04.017. 15220006

[50] Skaug B, Jiang X, Chen ZJ (2009) The role of ubiquitin in NF-κB regulatory pathways. Annu Rev Biochem 78: 769–796 10.1146/annurev.biochem.78.070907.102750 19489733

[51] Hoene M, Weigert C (2010) The stress response of the liver to physical exercise. Exercise immunology review 16: 163–183. 20839498

[52] Kjær M, Langberg H, Heinemeier K, Bayer ML, Hansen M, et al (2009) From mechanical loading to collagen synthesis, structural changes and function in human tendon. Scand J Med Sci Sports 19: 500–510 10.1111/j.1600-0838.2009.00986.x 19706001

[53] Matsakas A, Patel K (2009) Skeletal muscle fibre plasticity in response to selected envrionmental and physiological stimuli. Histol Histopathol 24: 209–222. 19283669 10.14670/HH-24.611

[54] Oh-hora M (2009) Calcium signaling in the development and function of T-lineage cells. Immunol Rev 231: 210–224 10.1111/j.1600-065X.2009.00819.x 19754899

[55] Bopp T, Radsak M, Schmitt E, Schild H (2010) New strategies for the manipulation of adaptive immune responses. Cancer Immunology, Immunotherapy 59: 1443–1448 10.1007/s00262-010-0851-z 20361184 PMC11030961

[56] Walsh NP, Gleeson M, Shephard RJ, Gleeson M, Woods JA, et al (2011) Position statement. part one: Immune function and exercise. Exercise immunology review 17: 6–63. 21446352

[57] Tonevitsky AG, Maltseva DV, Abbasi A, Samatov TR, Sakharov DA, et al (2013) Dynamically regulated miRNA-mRNA networks revealed by exercise. BMC Physiology 13: 9. 24219008 10.1186/1472-6793-13-9 PMC3681679

[58] D’Amours D, Jackson SP (2002) The Mre11 complex: At the crossroads of DNA repair and checkpoint signalling. Nature Reviews Molecular Cell Biology 3: 317–327 10.1038/nrm805 11988766

[59] Stracker TH, Theunissen JWF, Morales M, Petrini JHJ (2004) The Mre11 complex and the metabolism of chromosome breaks: The importance of communicating and holding things together. DNA Repair 3: 845–854 10.1016/j.dnarep.2004.03.014 15279769

[60] Wu Y, Xiao S, Zhu XD (2007) MRE11–RAD50–NBS1 and ATM function as co-mediators of TRF1 in telomere length control. Nature structural & molecular biology 14: 832–840 10.1038/nsmb1286 17694070

[61] Zhong ZH, Jiang WQ, Cesare AJ, Neumann AA, Wadhwa R, et al (2007) Disruption of telomere maintenance by depletion of the MRE11/RAD50/NBS1 complex in cells that use alternative lengthening of telomeres. J Biol Chem 282: 29314–29322 10.1074/jbc.M701413200 17693401

[62] Fumagalli M, Rossiello F, Clerici M, Barozzi S, Cittaro D, et al (2012) Telomeric DNA damage is irreparable and causes persistent DNA-damage-response activation. Nat Cell Biol 14: 355–365 10.1038/ncb2466 22426077 PMC3717580

[63] Von Zglinicki T (2002) Oxidative stress shortens telomeres. Trends Biochem Sci 27: 339–344 10.1016/S0968-0004(02)02110-2 12114022

[64] O’Connor MS, Safari A, Liu D, Qin J, Songyang Z (2004) The human Rap1 protein complex and modulation of telomere length. Journal of Biological Chemistry 279: 28585–28591 10.1074/jbc.M312913200 15100233

[65] Li B, Oestreich S, de Lange T (2000) Identification of human Rap1: Implications for telomere evolution. Cell 101: 471–483 10.1016/S0092-8674(00)80858-2 10850490

[66] Sfeir A, Kabir S, van Overbeek M, Celli GB, de Lange T (2010) Loss of Rap1 induces telomere recombination in the absence of NHEJ or a DNA damage signal. Science 327: 1657–1661 10.1126/science.1185100 20339076 PMC2864730

[67] Sarthy J, Bae NS, Scrafford J, Baumann P (2009) Human RAP1 inhibits non-homologous end joining at telomeres. EMBO J 28: 3390–3399 10.1038/emboj.2009.275 19763083 PMC2776107

[68] Martínez P, Gómez-López G, García F, Mercken E, Mitchell S, et al (2013) RAP1 protects from obesity through its extratelomeric role regulating gene expression. Cell Reports 3: 2059–2074 10.1016/j.celrep.2013.05.030 23791526 PMC5889507

[69] Platt JM, Ryvkin P, Wanat JJ, Donahue G, Ricketts MD, et al (2013) Rap1 relocalization contributes to the chromatin-mediated gene expression profile and pace of cell senescence. Genes Dev 27: 1406–1420 10.1101/gad.218776.113 23756653 PMC3701195

[70] Teo H, Ghosh S, Luesch H, Ghosh A, Wong ET, et al (2010) Telomere-independent Rap1 is an IKK adaptor and regulates NF-[kappa] B-dependent gene expression. Nat Cell Biol 12: 758–767 10.1038/ncb2080 20622870

[71] Barsov EV (2011) Telomerase and primary T cells: Biology and immortalization for adoptive immunotherapy. Immunotherapy 3: 407–421 10.2217/imt.10.107 21395382 PMC3120014

[72] Luiten RM, Pene J, Yssel H, Spits H (2003) Ectopic hTERT expression extends the life span of human CD4 helper and regulatory T-cell clones and confers resistance to oxidative stress-induced apoptosis. Blood 101: 4512–4519. 12586632 10.1182/blood-2002-07-2018

[73] Roth A, Yssel H, Pene J, Chavez EA, Schertzer M, et al (2003) Telomerase levels control the lifespan of human T lymphocytes. Blood 102: 849–857 10.1182/blood-2002-07-2015 12689947

[74] Rufer N, Migliaccio M, Antonchuk J, Humphries RK, Roosnek E, et al (2001) Transfer of the human telomerase reverse transcriptase (TERT) gene into T lymphocytes results in extension of replicative potential. Blood 98: 597–603 10.1182/blood.V98.3.597 11468156

[75] Weng NP (2001) Interplay between telomere length and telomerase in human leukocyte differentiation and aging. J Leukoc Biol 70: 861–867. 11739547

[76] Broccoli D, Young JW, de Lange T (1995) Telomerase activity in normal and malignant hematopoietic cells. Proceedings of the National Academy of Sciences 92: 9082–9086. 10.1073/pnas.92.20.9082 PMC40928 7568077

[77] Bestilny LJ, Brown CB, Miura Y, Robertson LD, Riabowol KT (1996) Selective inhibition of telomerase activity during terminal differentiation of immortal cell lines. Cancer Res 56: 3796–3802. 8706026

[78] Holt SE, Wright WE, Shay JW (1996) Regulation of telomerase activity in immortal cell lines. Molecular and Cellular Biology 16: 2932–2939. 8649404 10.1128/mcb.16.6.2932 PMC231287

[79] Epel ES, Lin J, Dhabhar FS, Wolkowitz OM, Puterman E, et al (2010) Dynamics of telomerase activity in response to acute psychological stress. Brain Behav Immun 24: 531–539 doi 10.1016/j.bbi.2009.11.018. 20018236 PMC2856774

[80] Gizard F, Heywood EB, Findeisen HM, Zhao Y, Jones KL, et al (2011) Telomerase activation in atherosclerosis and induction of telomerase reverse transcriptase expression by inflammatory stimuli in macrophages. Arteriosclerosis, Thrombosis, and Vascular Biology 31: 245–252 10.1161/ATVBAHA.110.219808 PMC3025413 21106948

[81] Hara H, Yamashita K, Shinada J, Yoshimura H, Kameya T (2001) Clinicopathologic significance of telomerase activity and hTERT mRNA expression in non-small cell lung cancer. Lung Cancer 34: 219–226 doi 10.1016/S0169-5002(01)00244-6. 11679180

[82] Kirkpatrick KL, Clark G, Ghilchick M, Newbold RF, Mokbel K (2003) hTERT mRNA expression correlates with telomerase activity in human breast cancer. European Journal of Surgical Oncology (EJSO) 29: 321–326 10.1053/ejso.2002.1374 12711283

[83] Saretzki G, Petersen S, Petersen I, Kölble K, von Zglinicki T (2002) hTERT gene dosage correlates with telomerase activity in human lung cancer cell lines. Cancer Lett 176: 81–91 doi 10.1016/S0304-3835(01)00644-9. 11790457

[84] Ulaner GA, Hu JF, Vu TH, Giudice LC, Hoffman AR (1998) Telomerase activity in human development is regulated by human telomerase reverse transcriptase (hTERT) transcription and by alternate splicing of hTERT transcripts. Cancer Research 58: 4168–4172. 9751630

[85] Liu K, Schoonmaker MM, Levine BL, June CH, Hodes RJ, et al (1999) Constitutive and regulated expression of telomerase reverse transcriptase (hTERT) in human lymphocytes. Proceedings of the National Academy of Sciences 96: 5147–5152 10.1073/pnas.96.9.5147 PMC21831 10220433

[86] Liu K, Hodes RJ, Weng N (2001) Cutting edge: Telomerase activation in human T lymphocytes does not require increase in telomerase reverse transcriptase (hTERT) protein but is associated with hTERT phosphorylation and nuclear translocation. The Journal of Immunology 166: 4826–4830. 11290757 10.4049/jimmunol.166.8.4826

[87] Yan P, Benhattar J, Coindre JM, Guillou L (2002) Telomerase activity and hTERT mRNA expression can be heterogeneous and does not correlate with telomere length in soft tissue sarcomas. International Journal of Cancer 98: 851–856 10.1002/ijc.10285 11948462

[88] Metzger R, Vallbohmer D, Müller-Tidow C, Higashi H, Bollschweiler E, et al (2009) Increased human telomerase reverse transcriptase (hTERT) mRNA expression but not telomerase activity is related to survival in curatively resected non-small cell lung cancer. Anticancer Research 29: 1157–1162. 19414359

[89] Swiggers SJ, Nibbeling HA, Zeilemaker A, Kuijpers MA, Mattern KA, et al (2004) Telomerase activity level, but not hTERT mRNA and hTR level, regulates telomere length in telomerase-reconstituted primary fibroblasts. Exp Cell Res 297: 434–443 doi 10.1016/j.yexcr.2004.03.028. 15212946

[90] Laye MJ, Solomon TPJ, Karstoft K, Pedersen KK, Nielsen SD, et al (2012) Increased shelterin mRNA expression in peripheral blood mononuclear cells and skeletal muscle following an ultra-long-distance running event. Journal of Applied Physiology 112: 773–781 10.1152/japplphysiol.00997.2011 22162529

[91] Gabriel H, Schmitt B, Urhausen A, Kindermann W (1993) Increased CD45RA+CD45RO+ cells indicate activated T cells after endurance exercise. Med Sci Sports Exerc 25: 1352–1357. 8107541

[92] Kruger K, Mooren FC (2007) T cell homing and exercise. Exerc Immunol Rev 13: 37–54. 18198659

[93] Bruunsgaard H, Jensen MS, Schjerling P, Halkjær-Kristensen J, Ogawa K, et al (1999) Exercise induces recruitment of lymphocytes with an activated phenotype and short telomeres in young and elderly humans. Life Sci 65: 2623–2633. 10619370 10.1016/s0024-3205(99)00531-7

[94] Simpson RJ, Cosgrove C, Chee MM, McFarlin BK, Bartlett DB, et al (2010) Senescent phenotypes and telomere lengths of peripheral blood T-cells mobilized by acute exercise in humans. Exerc Immunol Rev 16: 40–55. 20839490

[95] Campbell JP, Riddell NE, Burns VE, Turner M, van Zanten JJCS, et al (2009) Acute exercise mobilises CD8 T lymphocytes exhibiting an effector-memory phenotype. Brain Behav Immun 23: 767–775 doi 10.1016/j.bbi.2009.02.011. 19254756

[96] Aviv A, Valdes AM, Spector TD (2006) Human telomere biology: Pitfalls of moving from the laboratory to epidemiology. International Journal of Epidemiology 35: 1424–1429 10.1093/ije/dyl169 16997848

[97] Cawthon RM (2002) Telomere measurement by quantitative PCR. Nucl Acids Res 30: e47 10.1093/nar/30.10.e47 12000852 PMC115301

[98] Shen J, Terry MB, Gurvich I, Liao Y, Senie RT, et al (2007) Short telomere length and breast cancer risk: a study in sister sets. Cancer Research 67: 5538–5544 10.1158/0008-5472.CAN-06-3490 17545637

[99] Marfe G, Tafani M, Pucci B, Di Stefano C, Indelicato M, et al.. (2010) The effect of marathon on mRNA expression of anti-apoptotic and pro-apoptotic proteins and sirtuins family in male recreational long-distance runners. BMC physiology 10. doi: 10.1186/1472-6793-10-7. 10.1186/1472-6793-10-7 PMC2893521 20462402

[100] Haigis MC, Sinclair DA (2010) Mammalian sirtuins: Biological insights and disease relevance. Annual review of pathology 5: 253–295 10.1146/annurev.pathol.4.110807.092250 PMC2866163 20078221

[101] Mostoslavsky R, Chua KF, Lombard DB, Pang WW, Fischer MR, et al (2006) Genomic instability and aging-like phenotype in the absence of mammalian SIRT6. Cell 124: 315–329 10.1016/j.cell.2005.11.044 16439206

[102] McCord RA, Michishita E, Hong T, Berber E, Boxer LD, et al (2009) SIRT6 stabilizes DNA-dependent protein kinase at chromatin for DNA double-strand break repair. Aging (Albany NY) 1: 109–121. 20157594 10.18632/aging.100011 PMC2815768

[103] Mao Z, Hine C, Tian X, Van Meter M, Au M, et al (2011) SIRT6 promotes DNA repair under stress by activating PARP1. Science 332: 1443–1446 10.1126/science.1202723 21680843 PMC5472447

[104] Mauro C, Leow SC, Anso E, Rocha S, Thotakura AK, et al (2011) NF-[kappa] B controls energy homeostasis and metabolic adaptation by upregulating mitochondrial respiration. Nat Cell Biol 13: 1272–1279 10.1038/ncb2324 21968997 PMC3462316

[105] Kawahara TLA, Michishita E, Adler AS, Damian M, Berber E, et al (2009) SIRT6 links histone H3 lysine 9 deacetylation to NF-[kappa] B-dependent gene expression and organismal life span. Cell 136: 62–74 10.1016/j.cell.2008.10.052 19135889 PMC2757125

[106] Van Gool F, Gallí M, Gueydan C, Kruys V, Prevot PP, et al (2009) Intracellular NAD levels regulate tumor necrosis factor protein synthesis in a sirtuin-dependent manner. Nat Med 15: 206–210. 19151729 10.1038/nm.1906 PMC2845476

[107] Zhong L, D’Urso A, Toiber D, Sebastian C, Henry RE, et al (2010) The histone deacetylase Sirt6 regulates glucose homeostasis via Hif1 [alpha]. Cell 140: 280–293 10.1016/j.cell.2009.12.041 20141841 PMC2821045

[108] Effros RB (2011) Telomere/telomerase dynamics within the human immune system: Effect of chronic infection and stress. Exp Gerontol 46: 135–140 doi 10.1016/j.exger.2010.08.027. 20833238 PMC3246363

[109] van de Berg PJEJ, Griffiths SJ, Yong SL, Macaulay R, Bemelman FJ, et al (2010) Cytomegalovirus infection reduces telomere length of the circulating T cell pool. The Journal of Immunology 184: 3417–3423 10.4049/jimmunol.0903442 20176738

[110] Dowd JB, Bosch JA, Steptoe A, Blackburn EH, Lin J, et al (2013) Cytomegalovirus is associated with reduced telomerase activity in the whitehall II cohort. Exp Gerontol 48: 385–390 doi 10.1016/j.exger.2013.01.016. 23403382 PMC3626117

[111] Seale H, MacIntyre CR, Gidding HF, Backhouse JL, Dwyer DE, et al (2006) National serosurvey of cytomegalovirus in australia. Clinical and Vaccine Immunology 13: 1181–1184 10.1128/CVI.00203-06 16957061 PMC1656547

[112] Cannon MJ, Schmid DS, Hyde TB (2010) Review of cytomegalovirus seroprevalence and demographic characteristics associated with infection. Rev Med Virol 20: 202–213 10.1002/rmv.655 20564615

