# Supplementary material for: Acute Exercise Leads to Regulation of Telomere-Associated Genes and MicroRNA Expression in Immune Cells
Source: PLoS One. 2014 Apr 21;9(4):e92088. doi: 10.1371/journal.pone.0092088 (PMC3994003; doi:10.1371/journal.pone.0092088)
Supplement: Table S1 — Selected miRNAs and their potential mRNA interactions. (DOCX) [file pone.0092088.s001.docx]

| **miRNA** | **Potential gene target** | **Gene symbol** | **Prediction database** |
| --- | --- | --- | --- |
| miR-181b | Telomerase reverse transcriptase | *TERT* | miRanda (microrna.org) |
| miR-186 | RAD50 homolog | *RAD50* | miRanda (microrna.org) |
|  | Telomeric repeat binding factor 2, interacting protein | *TERF2IP* | TargetScan |
|  | Sirtuin-6 | *SIRT6* | miRanda (microrna.org) |
| miR-96 | Telomeric repeat binding factor 2, interacting protein | *TERF2IP* | miRanda (microrna.org) |
| miR-15a | TATA-box binding-protein | *TBP* | miRanda (microrna.org) |
