# Supplementary material for: Acute Exercise Leads to Regulation of Telomere-Associated Genes and MicroRNA Expression in Immune Cells
Source: PLoS One. 2014 Apr 21;9(4):e92088. doi: 10.1371/journal.pone.0092088 (PMC3994003; doi:10.1371/journal.pone.0092088)
Supplement: Table S2 — Quantitative real-time PCR TaqMan microRNA assays. (DOCX) [file pone.0092088.s002.docx]

| **miRNA** | **Assay identification #** |
| --- | --- |
| RNU44 (control) | 001094 |
| RNU48 (control) | 001006 |
| miR-181b | 001098 |
| miR-186 | 000486 |
| miR-96 | 000434 |
| miR-15a | 000389 |
