# Supplementary material for: Acute Exercise Leads to Regulation of Telomere-Associated Genes and MicroRNA Expression in Immune Cells
Source: PLoS One. 2014 Apr 21;9(4):e92088. doi: 10.1371/journal.pone.0092088 (PMC3994003; doi:10.1371/journal.pone.0092088)
Supplement: Table S3 — Quantitative real-time PCR gene expression primers (SYBR Green chemistry) and conditions. (DOCX) [file pone.0092088.s003.docx]

| **Gene symbol** | **GenBank Accession #** | **Primer Sequence (5’ 🡪 3’)** | **Concentration (nM)** | **Product length** | **Annealing temperature** |
| --- | --- | --- | --- | --- | --- |
| ***GAPDH*** | NM_002046.3 | Fwd: CTTTTGCGTCGCCAGCCGAG  Rev: GCGCCCAATACGACCAAATCCG | 200 | 86 | 58°C |
| ***TERT*** | NM_001103376.1 | Fwd: TACGGCGACATGGAGAACAAG  Rev: GGGCATAGCTGAGGAAGGTTT | 500 | 127 | 58°C |
| ***SIRT6*** | NM_001193285.1 | Fwd: CCACCAAGCACGACCGCCAT  Rev: CGCCCTCTCCAGCACACGG | 200 | 128 | 58°C |
