# Supplementary material for: Acute Exercise Leads to Regulation of Telomere-Associated Genes and MicroRNA Expression in Immune Cells
Source: PLoS One. 2014 Apr 21;9(4):e92088. doi: 10.1371/journal.pone.0092088 (PMC3994003; doi:10.1371/journal.pone.0092088)
Supplement: Table S4 — Quantitative real-time PCR TaqMan gene expression assays. (DOCX) [file pone.0092088.s004.docx]

| **Gene symbol** | **GenBank Accession #** | **Assay Identification #** |
| --- | --- | --- |
| ***RAD50*** | [NM_005732.3](http://www.ncbi.nlm.nih.gov/nuccore/NM_005732.3) | Hs00990023_m1 |
| ***TERF2IP*** | [NM_018975.3](http://www.ncbi.nlm.nih.gov/nuccore/NM_018975.3) | Hs00430292_m1 |
| ***GAPDH*** | NM_002046.3 | Hs02758991_g1 |

Fwd 5’ (forward primer sequence), Rev 3’ (reverse primer sequence)
