# Supplementary material for: Acute Exercise Leads to Regulation of Telomere-Associated Genes and MicroRNA Expression in Immune Cells
Source: PLoS One. 2014 Apr 21;9(4):e92088. doi: 10.1371/journal.pone.0092088 (PMC3994003; doi:10.1371/journal.pone.0092088)
Supplement: Table S5 — Significantly regulated miRNAs detected in genome-wide microarray. (DOCX) [file pone.0092088.s005.docx]

| **Probeset ID** | **Fold Change** | **FDR*†***  **value** | **Probeset ID** | **Fold Change** | **FDR*†***  **value** |
| --- | --- | --- | --- | --- | --- |
| hsa-miR-1270 | -11.45 | 0.03 | hsa-miR-320a | -1.20 | 0.03 |
| hsa-miR-96 | -1.99 | 0.03 | hsa-miR-320b | -1.19 | 0.03 |
| hsa-miR-675* | -1.53 | 0.03 | hsa-miR-139-3p | -1.19 | 0.03 |
| hsa-miR-574-3p | -1.47 | 0.03 | hsa-miR-320e | -1.18 | 0.03 |
| hsa-miR-576-5p | -1.47 | 0.04 | hsa-miR-4323 | -1.17 | 0.03 |
| hsa-miR-934 | -1.43 | 0.03 | hsa-miR-320d | -1.16 | 0.03 |
| hsa-let-7d* | -1.41 | 0.04 | hsa-miR-146a | -1.11 | 0.03 |
| hsa-miR-2115 | -1.39 | 0.03 | hsa-miR-186 | 1.16 | 0.04 |
| hsa-miR-193b | -1.38 | 0.03 | hsa-miR-15a | 1.17 | 0.03 |
| hsa-miR-636 | -1.35 | 0.03 | hsv2-miR-H6 | 1.18 | 0.03 |
| hsa-miR-1229 | -1.35 | 0.03 | hsa-miR-28-5p | 1.21 | 0.04 |
| hsa-miR-129* | -1.35 | 0.03 | hsa-miR-4322 | 1.26 | 0.03 |
| hsa-miR-129-3p | -1.34 | 0.03 | hsa-miR-3648 | 1.27 | 0.04 |
| hsa-miR-877* | -1.34 | 0.03 | hsa-miR-23a* | 1.33 | 0.04 |
| hsa-miR-1227 | -1.32 | 0.03 | hsa-miR-181b | 1.36 | 0.04 |
| hsa-miR-125b | -1.31 | 0.03 | hsa-miR-454* | 1.42 | 0.03 |
| hsv2-miR-H20 | -1.31 | 0.04 | hsa-miR-363 | 1.46 | 0.03 |
| kshv-miR-K12-8* | -1.30 | 0.03 | hsa-miR-583 | 1.66 | 0.03 |
| hsa-miR-933 | -1.29 | 0.03 | kshv-miR-K12-6-5p | 1.66 | 0.03 |
| hsv2-miR-H7-3p | -1.29 | 0.03 | hsa-miR-518c* | 1.67 | 0.04 |
| hsa-miR-3613-3p | -1.27 | 0.03 | hsa-miR-1276 | 1.73 | 0.04 |
| hsa-miR-3940 | -1.26 | 0.04 | hsa-miR-200b* | 1.86 | 0.04 |
| hsa-miR-378 | -1.25 | 0.03 | hsa-miR-3677 | 1.98 | 0.04 |
| hsa-miR-1225-3p | -1.25 | 0.04 | hsa-miR-1250 | 2.02 | 0.04 |
| hsa-miR-92a | -1.22 | 0.04 | hsa-miR-873 | 2.19 | 0.03 |
| hsa-let-7f-1* | -1.22 | 0.04 | hsa-miR-4316 | 2.37 | 0.03 |
| hsv1-miR-H1* | -1.21 | 0.03 | hsa-miR-764 | 3.26 | 0.03 |
| hsa-miR-550a* | -1.21 | 0.04 | hsa-miR-3146 | 4.35 | 0.03 |

The miRNA prefix ‘hsa’ denotes human origin, ‘hsv’ denotes herpes simplex virus, and ‘kshv’ denotes Kaposi’s sarcoma-associated herpes virus.

*†* FDR: false discovery rate
