# Supplementary material for: Acute Exercise Leads to Regulation of Telomere-Associated Genes and MicroRNA Expression in Immune Cells
Source: PLoS One. 2014 Apr 21;9(4):e92088. doi: 10.1371/journal.pone.0092088 (PMC3994003; doi:10.1371/journal.pone.0092088)
Supplement: Table S6 — Differential regulation obtained using the TaqMan Telomere extension array. (DOCX) [file pone.0092088.s006.docx]

| **Gene Symbol** | **Fold Change Pre-Post** | **Fold Change Pre-60min Post** | **Fold Change Post-60min Post** | **Gene Symbol** | **Fold Change Pre-Post** | **Fold Change Pre-60min Post** | **Fold Change Post-60min Post** |
| --- | --- | --- | --- | --- | --- | --- | --- |
| ***ACTB*** | 1.00 | 1.15 | 1.15 | ***HNRNPF*** | 1.18 | -1.12 | -1.33 |
| ***B2M*** | 1.10 | -1.27 | -1.40 | ***MRE11A*** | 1.13 | 1.14 | 1.01 |
| ***RPLPO*** | -1.03 | -2.09 | -2.04 | ***NBN*** | -1.03 | 1.07 | 1.10 |
| ***HMBS*** | -1.08 | 1.07 | 1.15 | ***POT1*** | -1.08 | -1.12 | -1.04 |
| ***TBP*** | 1.04 | -1.39 | -1.44 | ***RAD50*** | 1.05 | -1.17 | -1.23 |
| ***PGK1*** | 1.00 | 1.10 | 1.10 | ***TERF1*** | -1.37 | 1.38 | 1.90 |
| ***UBC*** | -1.05 | -1.03 | 1.02 | ***TERF2*** | -1.19 | 1.19 | 1.41 |
| ***PPIA*** | 1.01 | -1.46 | -1.48 | ***TERF2IP*** | 1.02 | -1.41 | -1.43 |
| ***TFRC*** | 1.03 | -1.13 | -1.16 | ***TERT*** | -1.14 | 1.93 | 2.20 |
| ***HNRNPA1*** | 1.17 | 1.31 | 1.12 | ***TINF2*** | 1.24 | 1.31 | 1.06 |
| ***HNRNPA2B1*** | 1.26 | 1.50 | 1.19 | ***TNKS*** | 1.02 | 1.52 | 1.49 |
| ***HNRNPAB*** | 1.13 | 1.06 | -1.07 | ***TNKS2*** | 1.11 | 1.31 | 1.18 |
| ***HNRNPC*** | 1.07 | 1.05 | -1.02 | ***XRCC5*** | 1.13 | -1.09 | -1.24 |
| ***HNRNPD*** | -1.26 | -1.19 | 1.06 | ***XRCC6*** | -1.02 | -1.02 | 1.00 |

All gene expression data is expressed relative to the average of four endogenous controls: *18S, GAPDH, HPRT1,* and *GUSB.* The TaqMan^®^ Human Telomere Extension array was only performed once due to limited sample, therefore results are limited to fold change without corresponding measures of statistical significance.
