# Supplementary material for: Acute Exercise Leads to Regulation of Telomere-Associated Genes and MicroRNA Expression in Immune Cells
Source: PLoS One. 2014 Apr 21;9(4):e92088. doi: 10.1371/journal.pone.0092088 (PMC3994003; doi:10.1371/journal.pone.0092088)
Supplement: Table S7 — Parallel regulation of miRNAs and corresponding potential target transcripts. (DOCX) [file pone.0092088.s007.docx]

| **miRNA** | **miRNA FC pre- to 60min post** | ***P* value** | **miRNA FC post- to 60min post** | ***P* value** | **Predicted target gene** | **Gene FC pre- to 60min post** | ***P* value** | **Gene FC post- to 60min post** | ***P* value** |
| --- | --- | --- | --- | --- | --- | --- | --- | --- | --- |
| miR-181b | 1.31 | *P* = 0.327 | 0.84 | *P* = 0.05 | *TERT* | 19.38 | *P* < 0.01 | 14.75 | *P* < 0.05 |
| miR-186 | 1.93 | *P* < 0.001 | 1.58 | *P* < 0.001 | *SIRT6* | 1.67 | *P* < 0.05 | 1.66 | *P* < 0.05 |
|  |  |  |  |  | *RAD50* | 0.91 | *P* = 1.0 | 0.76 | *P* = 0.05 |
|  |  |  |  |  | *TERF2IP* | 0.8 | *P* = 1.0 | 0.54 | *P* < 0.01 |
| miR-96 | 2.63 | *P* < 0.001 | 2.11 | *P* < 0.01 | *TERF2IP* | 0.8 | *P* = 1.0 | 0.54 | *P* < 0.01 |

FC = fold change
